# The Impact of Hydration and Temperature on Bacterial Diversity in Arid Soil Mesocosms

**DOI:** 10.3389/fmicb.2017.01078

**Published:** 2017-06-14

**Authors:** Adam Št'ovíček, Ani Azatyan, M. Ines M. Soares, Osnat Gillor

**Affiliations:** Zuckerberg Institute for Water Research, Blaustein Institutes for Desert Research, Ben-Gurion University of the NegevMidreshet Ben Gurion, Beersheba, Israel

**Keywords:** bacteria, desert soil, hydration, desiccation, mesocosm

## Abstract

Hot desert ecosystems experience rare and unpredictable rainfall events that resuscitate the arid flora and fauna. However, the effect of this sudden abundance of water on soil microbial communities is still under debate. We modeled varying rainfall amounts and temperatures in desert soil mesocosms and monitored the microbial community response over a period of 21 days. We studied two different wetting events, simulating heavy (50 mm) and light (10 mm) rain, as well as three different temperature regimes: constant 25° or 36°C, or a temperature diurnal cycle alternating between 36 and 10 °C. Amplicon sequencing of the bacterial ribosomal RNA revealed that rain intensity affects the soil bacterial community, but the effects are mitigated by temperature. The combination of water-pulse intensity with lower temperature had the greatest effect on the bacterial community. These experiments demonstrated that the soil microbial response to rain events is dependent not only on the intensity of the water pulse but also on the ambient temperature, thus emphasizing the complexity of bacterial responses to highly unpredictable environments.

## Introduction

Hot arid and hyper-arid ecosystems stretch across more than 30% of the Earth's surface, making them the largest land mass biome. An increasing number of countries are struggling with an ever-increasing rate of desertification and water scarcity (Bestelmeyer et al., 2015). Understanding the dynamics of desert ecology is essential to challenging the accelerated desertification process.

For most of the year, arid areas experience severe water shortage, interrupted by brief and highly variable precipitation pulses (Noy-Meir, 1973). These rain events supply the main limiting resource, water, resuscitating dormant annuals and reviving water-limited perennial shrubs (Austin et al., 2004; Chesson et al., 2004). While the desert flora and fauna responses to rainfall events have been relatively well described, the reaction of soil microorganisms to water pulses remains unclear. Some studies have reported large-scale metabolic activation upon wetting of the soil following prolonged draught (Fierer and Schimel, 2003; Huxman et al., 2004; Borken and Matzner, 2009), pointing to dramatic changes in microbial diversity and community composition (Placella et al., 2012; Angel and Conrad, 2013).

The frequencies of seasonal rain events have been shown to be closely related to microbial activity. Prolonged droughts affect microbial activity and metabolism, whereas rain events entail changes in microbial community composition and function (Schimel et al., 2007; Collins and Sinsabaugh, 2008). Changes in the microbial community upon soil hydration have been proposed to be successive, featuring the emergence and decline of various taxa, starting with an increase in Firmicutes, shortly after the rain, followed by Proteobacteria (mainly α, β, and γ) and a rapid decline of Actinobacteria (Placella et al., 2012). These changes have been attributed to hydration-mediated competition (Dechesne et al., 2010; Wang and Or, 2012, 2013), predation (Schnürer et al., 1986), or phage bloom (Ashelford et al., 2003) instigated by aqueous connectivity of the soil microhabitats. Understanding the parameters governing the impact of hydration–desiccation cycles in desert soil on the microbial community is essential to modeling climatic changes and their effect on soil functions.

In this study, we tested the response of desert soil bacterial communities to hydration and desiccation cycles by manipulating rain intensity simulating light rain and heavy rain and incubation conditions (applying mild and hot temperatures as well as diurnal cycles). We hypothesized that shifts in arid soil bacterial communities would be primarily governed by rain intensity and to a lesser extent, by ambient temperature. We further predicted that temperature diurnal cycles rather than constant incubation temperature would contribute to alterations of the soil microbial communities. To test our predictions, we set up a desert soil mesocosm equipped with rain simulators and examined hydration–desiccation cycles under different rain intensities and temperatures.

**Figure 1:**
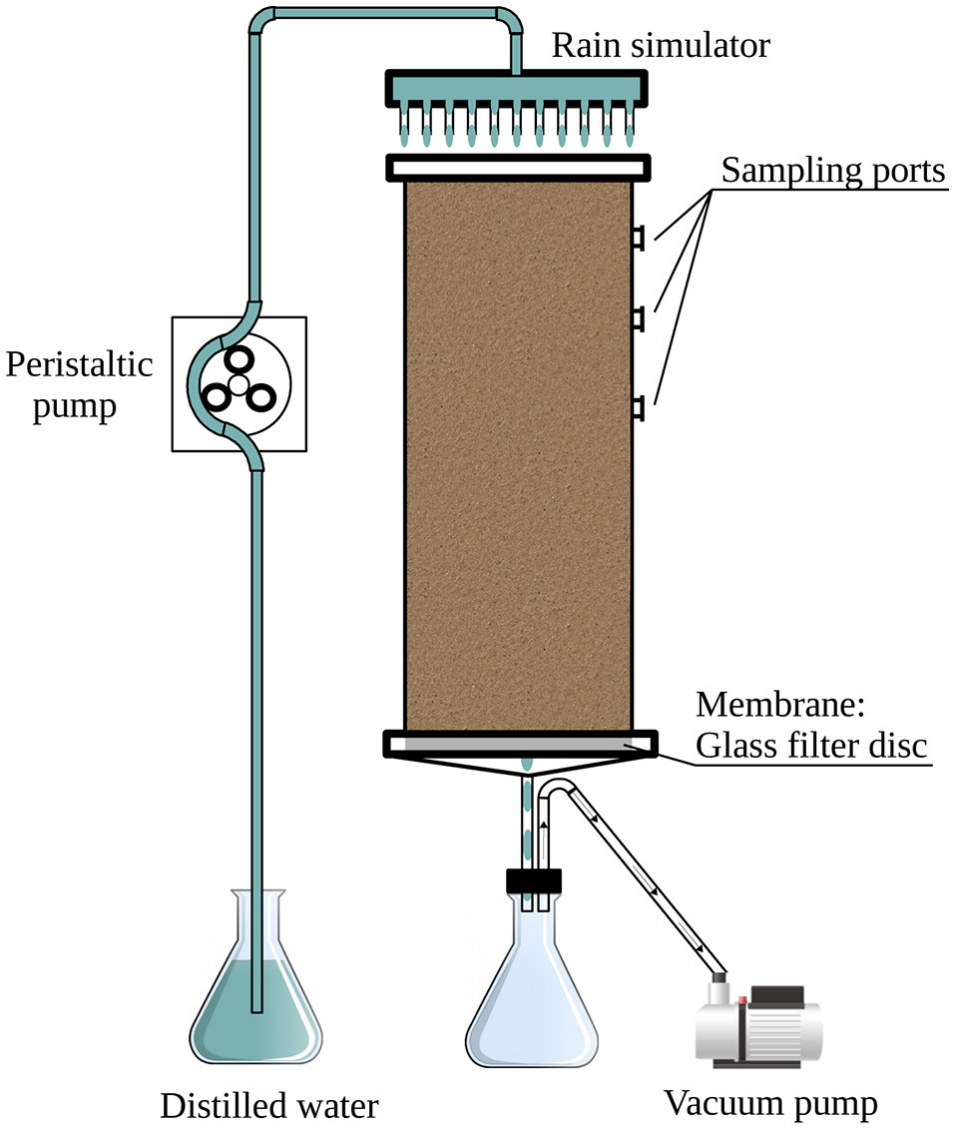
Schematic representation of the mesocosm setup used in this study.

In this study, controlled experiments were used to explore the effects of rain intensity and ambient temperature on the bacterial community in desert soil. The soil was collected during the summer months when the water content was minimal. We collected topsoil, devoid of crust, and packed it into mesocosms (Figure 1). In this way, all observed effects were decoupled from the crust's primary producers (Lange et al., 1992; Garcia-Pichel and Belnap, 1996). This approach corresponds with previously reported systems used for studying the response of arid soil microbial communities to rain events (Fierer and Schimel, 2003; Placella et al., 2012; Frossard et al., 2015), rendering our results comparable. In addition, the system used here has the advantage of uniformity, as the soil was well homogenized thus limiting confounding factors that might have otherwise affected the experiments. Lastly, in this study, we used, for the first time, soil mesocosms equipped with a rain simulator designed to better simulate field conditions.

## Materials and methods

### Soil collection and processing

The soil used in the experiments was collected from barren patches of an unmarked plot at the long-term ecological research (LTER) station of Avdat (30°47' N, 34°46' E; 600–700 m elevation) during the summer of 2012. The mean annual rainfall in the region is ~90–130 mm and rain occurs only during the winter months (UNESCO, 1979). Eight randomly selected subsamples were taken from the top 5 cm of the bulk soil (using an ethanol-cleaned scoop), after removing the crust. The collected soil was placed in sterile bags (Whirl-Pack, Nasco, Fort Atkinson, WI), transported to the laboratory and kept at 4°C until its homogenization, within less than 24 h, by passing through an autoclaved 2-mm grid size sieve. Subsamples were taken for physicochemical analyses and a total of 3.3 kg of homogenized soil was packed in each of three replicate columns (Figure 1).

### Experimental design

A full factorial design was used to evaluate the soil microbial community response to hydration–desiccation cycles, resulting in 72 samples (3 replicate mesocosms × 4 treatments × 6 sampling times). The mesocosms (Figure 1) consisted of three soil-packed polyvinyl chloride (PVC) cylinders (30 cm high, 10 cm in diameter) fitted at the bottom with a 4- to 8-μm pore size ceramic filter (Ace Glass, Vineland, NJ, USA). An outlet tube at the base of the column led to a continuously operating vacuum pump to simulate natural capillary forces in the soil. Ten sampling ports were located around the top 12 cm of the column; the ports were fitted with Suba-Seal rubber septa (Sigma-Aldrich, St. Louis, MO, USA).

Rain events were mimicked with a shower-like rain simulator (Figure 1) consisting of a PVC disk (10 cm in diameter) equipped with 21 syringe needles (0.4 × 13 mm) dripping double-distilled water (DDW) onto the soil surface. The simulated rain was dispersed at a rate of 1.5 ml min^−1^ by means of a peristaltic pump (Gilson Minipuls 3, Middleton, WI, USA). The applied volume of water was 80 or 400 ml, corresponding to light and heavy rain, respectively. Following the single rain event, the columns were kept in the dark for 21 days under constant temperature of 25 or 36°C, or under a 12 h diurnal cycle of 36 and 10°C (Table 1).

**Table 1:** Conditions applied to the soil columns and estimated average water loss during the 21 days experiments.

**Treatment**	**Temperature (°C)**	**Rain event (mm)**
Heavy rain and lower temperature	25	50
Light rain and lower temperature	25	10
Heavy rain and temperature diurnal cycle	36/10	50
Heavy rain and high temperature	36	50

### Mesocosm sampling

The soil columns were sampled 0.5, 1.5, and 3 days after the rain event, and then once a week for up to 3 weeks. The samples were removed with a sterile spatula through the column side ports (Figure 1). A total of 45–60 g soil was removed from each column and divided, resulting in triplicate samples for each sampling time. Samples from the soil homogenate prior to packing into the columns were considered the baseline prior to treatment (time 0). For molecular analyses, 20–30 g of soil from each time point was suspended in RNAlater (Zoetendal et al., 2006) at a 1:1 ratio (w/v), for better stabilization of the cellular RNA, and stored at −80°C.

### Physicochemical analyses

Soil physicochemical properties were determined according to standard methods (Smith and Doran, 1996; Doran et al., 1996a,b). Soil water content was determined by gravimetry. Electrical conductivity (EC) and pH were measured with EC electrodes and pH meter, respectively, in saturated soil extract. Nitrite-N was determined by colorimetric method (HCl, sulfanilamide and ethylenediamine dihydrochloride mix) using an Infinite M200 spectrophotometer (Tecan, Männedorf, Switzerland). Nitrate-N was measured by the second-derivative method (Si et al., 2007) using a BioMate 5 spectrophotometer (Thermo Scientific, Waltham, MA, USA).

### RNA extraction and cDNA synthesis

Total nucleic acids were extracted as previously described (Angel, 2012). We followed bacterial RNA, which has been proven to be a better indicator of active community variations than DNA (Blazewicz et al., 2013). The obtained RNA was purified using an RNA purification kit (Epicenter, Madison, WI, USA) according the manufacturer's instructions. The RNA was reverse-transcribed to cDNA using ImProm-II™ Reverse Transcriptase (Promega, Madison, WI, USA) in the presence of Recombinant RNasin Ribonuclease Inhibitor (Promega) following the manufacturer's protocol. The resulting cDNA was purified by PCR purification kit (Bioneer, Daejeon, Republic of Korea) and quantified spectrophotometrically with Nanodrop (Thermo Scientific).

### Bacterial abundance

The abundance of total bacteria, *Actinobacteria* and *Firmicutes* populations was quantified using group-specific quantitative (q) PCR assays targeting the 16S ribosomal (r) RNA units (Supplementary Table 1). All qPCRs were performed in an iCycler thermocycler equipped with a MyiQ detection system (Bio-Rad, Munich, Germany), and the data were processed using Bio-Rad CFX Manager 3.0 software. The quantification assays were based on SYBR Green I quantification (Thermo Scientific).

For all assays, standards containing known copy numbers of the target gene were used. Genomic DNA of *Streptomyces griseus* was used to quantify total bacterial and *Actinobacteria*. Cloned fragment of the 16S rRNA-encoding genes obtained from *Bacillus subtilis* was used to quantify the *Firmicutes* abundance. To evaluate total bacteria each qPCR contained 10 μl SYBR Absolute Blue qPCR Rox Mix (Thermo Scientific), 1 μl of 400 nM of each primer (Metabion, Munich, Germany), 5 μl template cDNA and 3 μl molecular-grade water (HyLab, Rehovot, Israel). To quantify the *Firmicutes* population, each qPCR contained 12.5 μl SYBR Absolute Blue qPCR Rox Mix, 0.5 μl of 250 nM of each primer, 5 μl template, and 6.5 μl molecular-grade water. Each reaction was repeated at least twice.

Bacterial abundance was estimated under the following conditions: 95°C for 15 min, followed by 35 cycles of 95°C for 10 s, 60°C for 15 s and 72°C for 30 s for extension. Conditions for estimating Actinobacteria abundance were: 95°C for 15 min, followed by 35 cycles of 95°C for 45 s, 63°C for 45 s and 72°C for 45 s. Firmicutes abundance estimation conditions were: 95°C for 15 min, followed by 35 cycles of 95°C for 10 s, 65°C for 15 s and 72°C for 45 s.

### qPCR data analysis

The effect of changing soil physicochemical parameters during each of the experiments was studied with linear mixed models using the R package lme4 v1.1-12 (Bates et al., 2015). The different experimental conditions were modeled together, each with a random slope to account for a different scale of community response to each condition. *P*-values were estimated using the Satterthwaite approximation for degrees of freedom. More information about the statistical analyses can be found in the Supplementary Information: qPCR model.

### Sequencing analysis

Amplicons of the soil bacterial 16S rRNA regions V3 and V4 were sequenced at the Core Facility of the University of Illinois (http://www.rrc.uic.edu/). The generated reads were quality-filtered and checked for chimeric sequences using the usearch method (Edgar, 2010). Sequences were aligned with the PyNAST tool (Caporaso et al., 2010a) and clustered into operational taxonomic units at 90% similarity between 16S rRNA using an open reference picking pipeline provided by the Qiime package (Caporaso et al., 2010b). Operational taxonomic units were picked and analyzed using Silva 111 (Quast et al., 2013) and sequences that were not present in the database were clustered *de novo*. The number of sequences in each sample was randomly subset (rarefied) to 9,000 sequences per sample using the phyloseq package (McMurdie and Holmes, 2013; Oksanen et al., 2016). Operational taxonomic units appearing more than 500 times in the data set were selected for a community structure stack plot and averaged per putative cluster. Both total counts were then normalized to 1.

### Diversity analyses

Evenness and richness of the soil bacterial community were studied during each experiment. We chose species count as representative of population richness and Pielou's measure of species evenness as an estimator of population shape (Pielou, 1966).

### Statistical analysis of the effect of chemical data on the community

The effect of the measured soil physicochemical parameters on community richness and evenness was examined. The overall effect was evaluated using a linear mixed model with random effects from the R package lme4 v1.1-12 (Bates et al., 2015). A random slope was introduced for the different experimental conditions. Details of the analysis can be found in the Supplementary Information: Evenness model, qPCR model, Richness model and Canonical correspondence analysis (CCA) model. *P*-values were estimated using the Satterthwaite approximation for degrees of freedom.

## Results

Soil mesocosms were designed to assess the effects of rain intensity and temperature on the bacterial community in desert soil (Figure 1). The mesocosms were sampled for a period of 3 weeks following the simulated rain event to assess the changes in soil physicochemical properties and bacterial abundance, diversity and community composition.

### Soil physicochemical parameters

**Figure 2:**
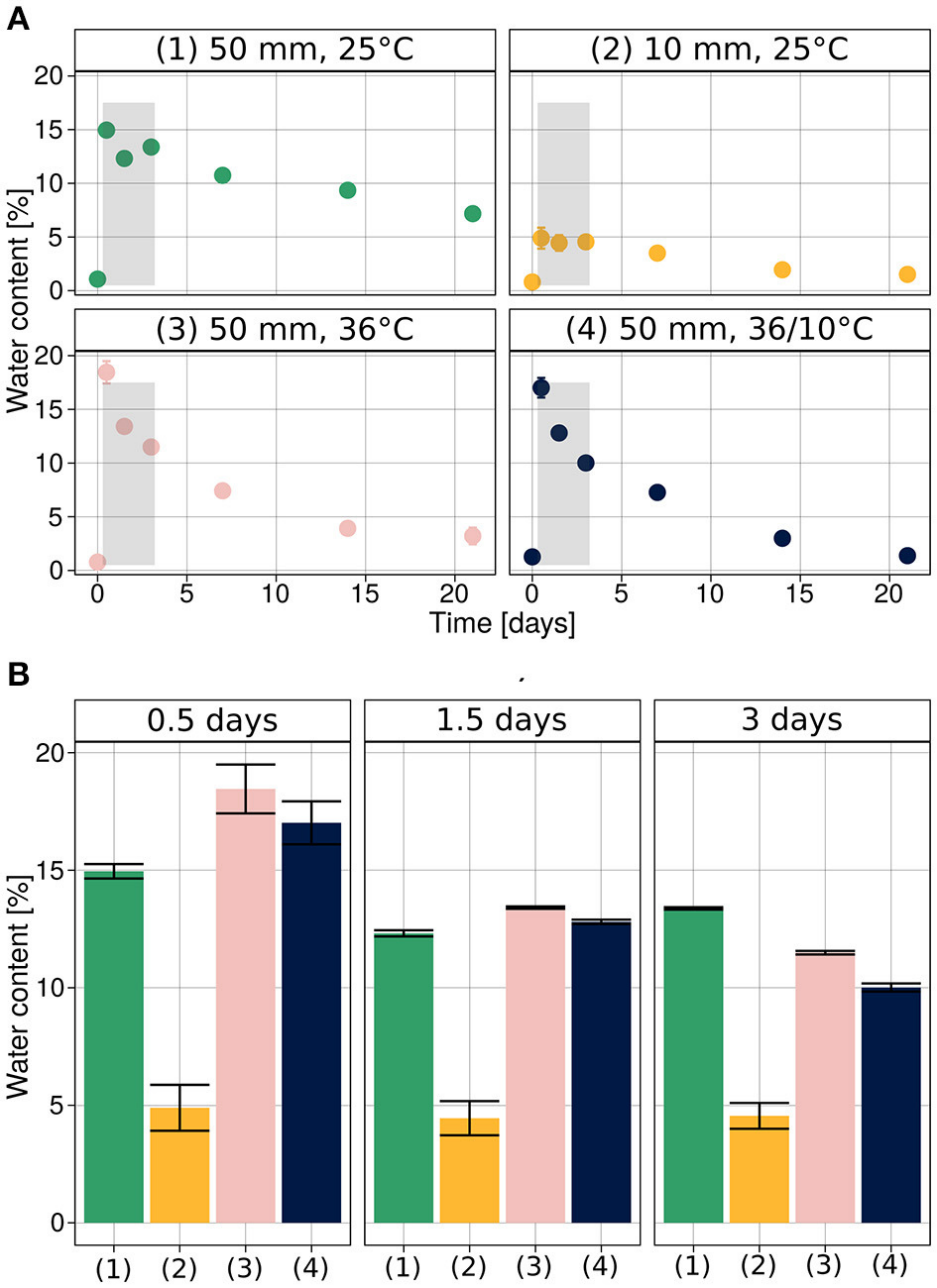
Soil water content during hydration–desiccation. **(A)** Under each of the experimental conditions. **(B)** Direct comparison of the four treatments for the first three sampling times after wetting. Average values are presented as mean ± SEM, based on 12 observations per data point.

The initial water content in the soil was very low, 0.97% ± 0.05 (SEM). It increased, following the rain simulations, to 16.72% ± 0.5 after the heavy rain (50 mm), corresponding to field saturation of loess soil (Muñoz-Castelblanco et al., 2012), and 4.89% ± 0.98 after the light rain (10 mm) (Figure 2A).

We note that during the initial stages of the experiments (0.5, 1.5, and 3 days after the rain event), the soil water content was similar when heavy rain was applied (Figure 2B), despite the different temperatures. At later stages (1, 2, and 3 weeks after hydration), the higher temperature and diurnal cycle conditions increased desiccation of the soil mesocosm.

The salinity (EC) of the soil dropped in the early stages of hydration and increased at the later stages of desiccation, as the capillary rise transferred salts back to the sampled level of the mesocosms. This effect was less pronounced when heavy rain and lower temperature were applied, probably because the soil did not fully desiccate (Supplementary Table 2). Under all conditions tested, soil pH was stable at around 8.5.

The concentration of ammonium-N increased after both heavy and light rain events when the temperature was lower (Supplementary Table 2). The elevated concentration of ammonium-N under higher temperature and diurnal cycles might have been due to external contamination of the samples; however, during the experiments, the concentrations of ammonium equilibrated under both conditions (Supplementary Table 2).

The concentrations of nitrite- and nitrate-N did not change markedly during the experiments. Nevertheless, under higher temperature and diurnal cycles, elevated concentrations were found in the initial measurements, possibly due to external contamination. Nitrate-N concentration decreased during the early stages of the experiments, returning to its original value in the later stages of each experiment (Supplementary Table 2).

### Dynamics of bacterial abundance during hydration–desiccation

**Figure 3:**
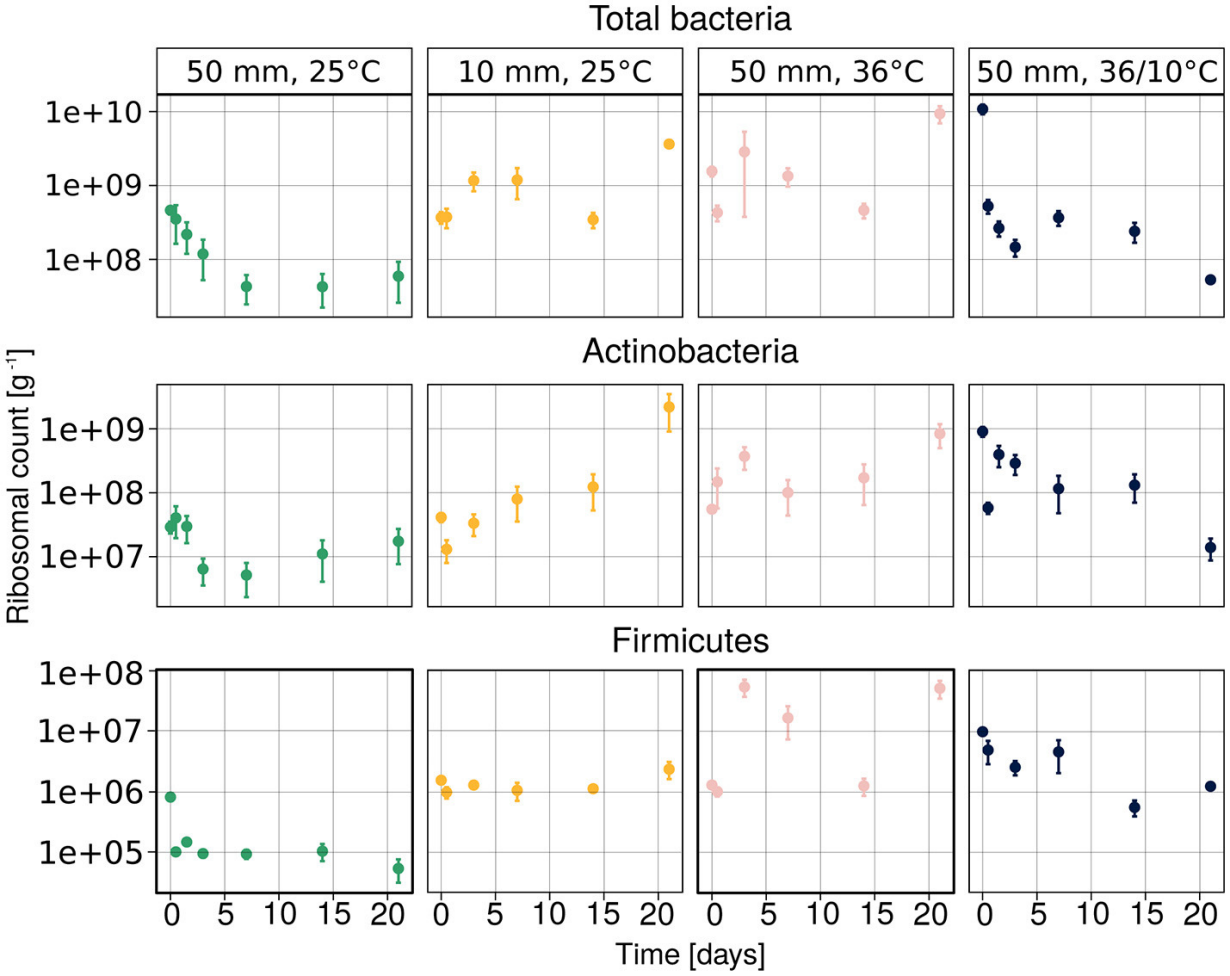
Abundance of total bacteria, *Actinobacteria* and *Firmicutes* ribosomes, measured by the qPCR method. Average values of three samples are presented as mean ± SEM.

The temporal changes in the abundance of total bacteria, Actinobacteria and Firmicutes, as assessed by amplification and detection of the 16S rRNA by qPCR, are illustrated in Figure 3. The results suggest that the quantity of the bacterial 16S ribosomes were strongly dependent on treatment. The ribosomal abundance of total soil bacteria, Actinobacteria and Firmicutes decreased after heavy rain at the lower temperature, and then remained low throughout the experiment. No clear trend could be observed under the other three sets of conditions, except for the abundance of Actinobacteria, which increased over time under light rain at the lower temperature.

According to the linear mixed model, the different temperature conditions had the strongest effect on each of the observed groups (Table 2). The soil concentration of ammonium-N had a significant effect on the abundance of total bacteria (*t* = 3.615, *p* = 0.0006) as well as on that of Actinobacteria (*t* = 2.101, *p* = 0.039). The soil water content had a significant effect on total bacterial population (*t* = −2.137, *p* = 0.036).

**Table 2:** Results of linear mixed model of the effects of the measured physicochemical parameters and sampling time on community richness and evenness.

**Pielou Evenness**					
**RANDOM EFFECTS**					
**Groups**	**Name**	**Variance**	**SD**		
Experiment	(Intercept)	0.0007176	0.02679		
Residual		0.0006894	0.02626		
Number of observations: 37, groups: 4					
**FIXED EFFECTS**					
	**Estimate**	**SE**	**Df**	***t*****-value**	**Pr(>|t|)**
(Intercept)	0.6102468	0.0182342	7.810	33.467	1.03e-09^***^
Time	−0.0010238	0.0007006	37.880	−1.461	0.152
Water content	−0.0015646	0.0010888	38.550	−1.437	0.159
**Richness**					
**RANDOM EFFECTS**					
**Groups**	**Name**	**Variance**	**SD**		
Experiment	(Intercept)	12,469	111.67		
Residual		5,618	74.95		
Number of observations: 36, groups: 5					
**FIXED EFFECTS**					
	**Estimate**	**SE**	**df**	***t*****-value**	**Pr(>|t|)**
(Intercept)	1,156.019	144.281	20.823	8.012	8.54e-08^***^
EC	−165.136	80.198	29.385	−2.059	0.048^*^
NO_2_-N	294.375	131.227	30.178	2.242	0.032^*^
Time	−11.108	2.667	30.783	−4.166	0.0002^***^
Water content	−23.065	6.160	25.708	−3.762	0.0009^***^

Only the simplified model is presented. Significance codes:

*** p_r_ < 0.001; ^**^p_r_ < 0.01;

* *p_r_ < 0.05; ^.^p_r_ < 0.1*.

### Bacterial diversity

**Figure 4:**
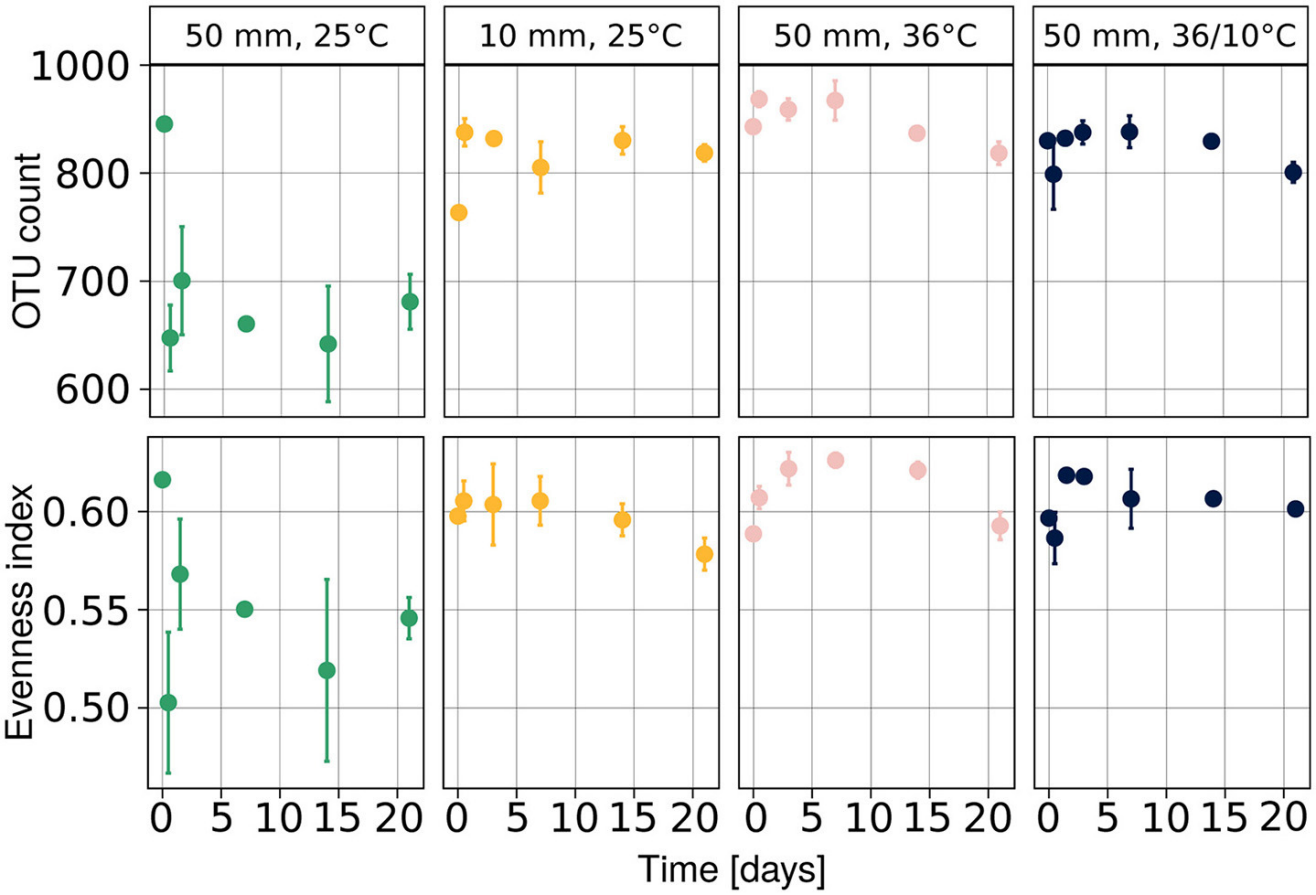
Diversity of the bacterial community at each time point expressed as evenness and richness components. Population richness is expressed as number of observed species and evenness was evaluated with Pielou's index. Average values are presented as mean ± SEM, based on three observations per data point. OTU, operational taxonomic units.

We observed a drop in both population richness and evenness (Figure 4) under heavy rain and lower ambient temperature, with neither of these two measures recovering for the duration of the experiment. In contrast, no marked differences were detected in richness or evenness under the other applied treatments (Figure 4), i.e., light rain, higher temperature or temperature diurnal cycles. The effect of soil chemical parameters on bacterial richness and evenness was analyzed by a linear mixed model with random effects (Table 3). The results suggest that sampling time, nitrite-N, EC, and water content had significant effects on both bacterial richness and evenness (Table 3).

**Table 3:** Effect of soil water content, ammonium-N concentration and sampling time on the abundance of ribosomes of total bacteria, *Actinobacteria* and *Firmicutes*.

**Total bacteria**					
**RANDOM EFFECTS**					
**Groups**	**Name**	**Variance**	**SD**		
Experiment	(Intercept)	0.9739	0.9869		
Residual		2.1853	1.4783		
Number of observations: 73, groups: 4					
**FIXED EFFECTS**					
	**Estimate**	**SE**	**df**	***t*****-value**	**Pr(>|t|)**
(Intercept)	19.17112	0.62988	5.55	30.436	2.17E-07^***^
NH_4_-N	0.05024	0.01390	69.47	3.615	0.0006^***^
Water content	−0.08040	0.03763	69.98	−2.137	0.036^*^
***Actinobacteria***					
**RANDOM EFFECTS**					
Groups	Name	Variance	SD		
Experiment	(Intercept)	1.227	1.108		
Residual		4.181	2.045		
Number of observations: 73, groups: 4					
**FIXED EFFECTS**					
	**Estimate**	**SE**	**df**	***t*****-value**	**Pr(>|t|)**
(Intercept)	16.63714	0.77196	6.65	21.552	2.09e-07^***^
NH_4_-N	0.03988	0.01898	66.98	2.101	0.039^*^
Water content	−0.03995	0.05162	69.68	−0.774	0.44
***Firmicutes***					
**RANDOM EFFECTS**					
**Groups**	**Name**	**Variance**	**SD**		
Experiment	(Intercept)	1.854	1.362		
Residual		1.390	1.179		
Number of observations: 73, groups: 4					
**FIXED EFFECTS**					
	**Estimate**	**SE**	**df**	***t*****-value**	**Pr(>|t|)**
(Intercept)	13.48449	0.73155	3.44	18.433	0.00015^***^
NH_4_-N	0.02366	0.01304	49.27	1.185	0.076^.^

Simplified models are presented. Significance codes:

*** p_r_ < 0.001; ^**^p_r_ < 0.01;

* p_r_ < 0.05;

. *p_r_ < 0.1*.

**Figure 5:**
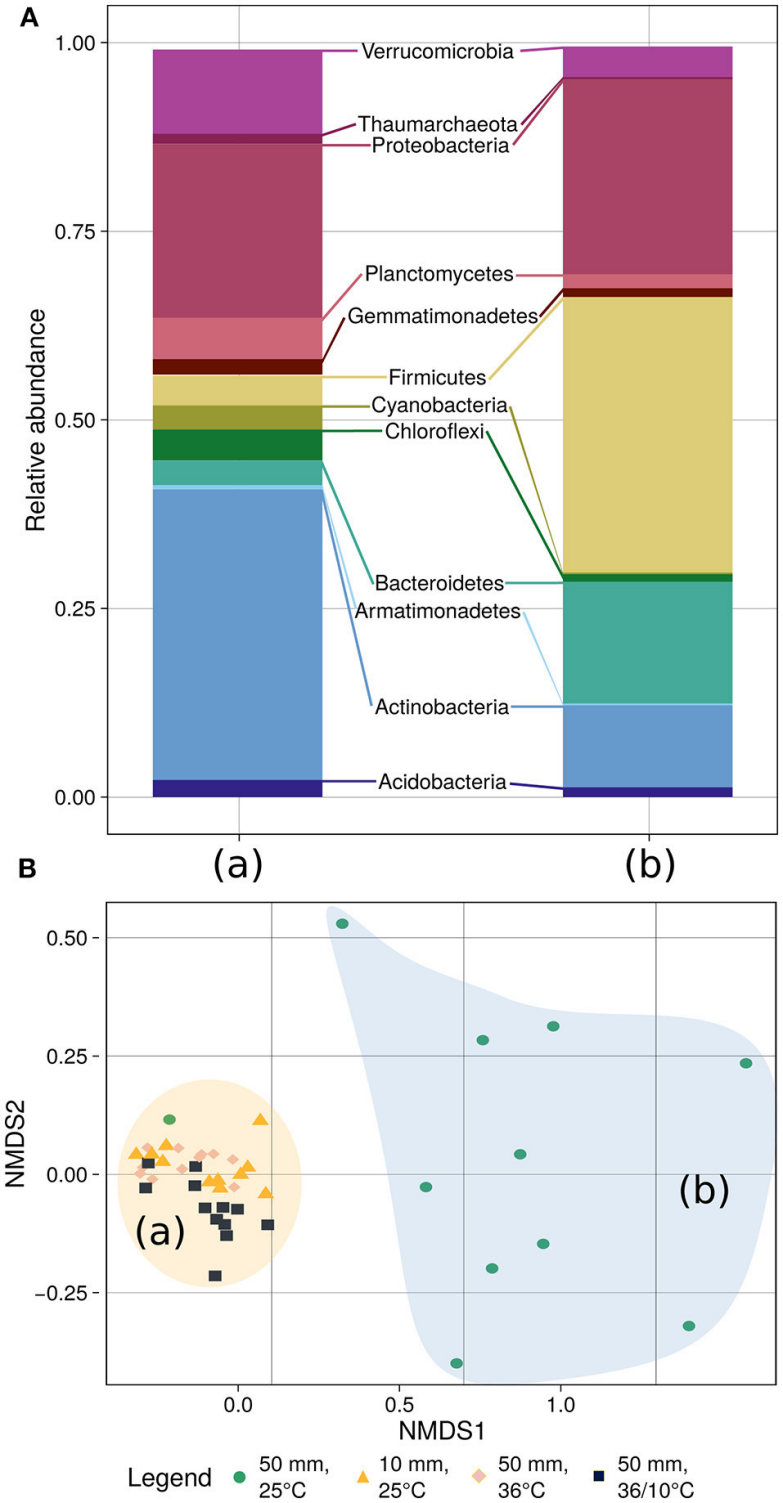
Community composition **(A)** and NMDS-based clustering analysis **(B)** of the bacterial population. **(A)** The community composition analysis differentiates between the soil bacteria response to heavy rain and lower temperature (right bar) and the other three experiments (left bar) where the bacterial community showed marginal differences between experiments (Supplementary Table 3). **(B)** The diversity analysis underlines the community composition results differentiating between the experiments. Stress of the dimensionality reduction is 0.083, meaning that the plot well represents the data set.

To further explore which members of the bacterial community changed during the experiments, we analyzed its composition and plotted the data using non-metric multidimensional scaling (NMDS), applying the Bray–Curtis distance measure (Figure 5). The final stress of the dimensionality reduction is equal to 0.083, suggesting that the plot well represents the data set. Further analysis using ANOSIM from the Vegan package (v2.4-1) in R (Oksanen et al., 2016) revealed that the community composition changed significantly during the experiment (*R* = 0.3797, *p* < 2.10^−5^, 50,000 permutations) (Supplementary Figure 1). These changes were probably due to the community shift upon application of heavy rain and lower temperature. However, when the soil was treated with light rain and lower ambient temperature, heavy rain and higher temperature, or heavy rain and a temperature diurnal cycle, no marked shifts in community composition were detected (Figure 5, Supplementary Figure 2). The phyla Actinobacteria, Bacteriodetes and Firmicutes showed the most significant differences between the “unchanged” cluster and the heavy rain and lower temperature cluster. Actinobacteria decreased from 39.0 to 11.3% under heavy rain and lower temperature, whereas Bacteroidetes increased from 3.3 to 16.4% and Firmicutes from 4.1 to 35.9% under these conditions. Details of community composition are presented in Supplementary Table 3.

## Discussion

The dynamic of bacterial communities in arid soil following a hydration and desiccation cycles is poorly understood (Št'ovíček et al., 2017). Following rain events in the desert, we anticipated a reduction in bacterial richness, considering previous reports of hydration-mediated soil connectivity proposed to result in competition (Dechesne et al., 2010; Wang and Or, 2012, 2013), phage blooms (Ashelford et al., 2003), and/or increased predation (Schnürer et al., 1986). However, we observed marked changes, only, in the soil bacterial community under heavy rain and lower temperature (Figure 3, Table 2), conditions that yielded a significant response in both the diversity (Figure 4) and composition (Figure 5) of the community. Under light rain the overall water content was low (Figure 2), and the bacterial diversity did not change (Figure 4), suggesting that the community more readily responded to heavy rain. Likewise, a yearlong study in the Namib Desert revealed that only a major rain event had dramatic effect on the microbial community composition (Armstrong et al., 2016). Yet, when heavy rain was applied while the temperatures were higher, the changes in soil bacterial community were suppressed (Figure 5, Supplementary Figure 2) suggesting that low incubation temperature in tandem with heavy rain are necessary to induce arid soil microbial community shifts.

Similar to the reduction in richness, we expected a reduction in total ribosomal quantity due to the hydration-mediated soil dynamics (Schnürer et al., 1986; Ashelford et al., 2003; Dechesne et al., 2010). However, similar to the diversity measures, this reduction occurred only under the low temperature and heavy rain conditions (Figure 3), mimicking the Negev Desert conditions during the wet season (Noy-Meir, 1973; Št'ovíček et al., 2017). The other applied environmental conditions did not result in marked decrease in the total ribosomal counts following hydration (Figure 3).

Alongside the total ribosomal count, we followed two selected bacterial taxa, Actinobacteria and Firmicutes, because they have been shown to be the first responders to soil wetting after a long drought (Cruz-Martinez et al., 2012; Placella et al., 2012; Angel and Conrad, 2013; Št'ovíček et al., 2017). Both taxa changed under heavy rain and the lower ambient temperature (Figure 3), following the changes in ammonium-N concentrations (Table 3, Supplementary Figure 2). It was previously suggested that the first rain after a long draft changes Mediterranean soil water potential triggering plant germination, litter decomposition, and microbial activity that quickly assimilate nitrogen (Cruz-Martinez et al., 2012). Yet, in this study the results are not conclusive (Supplementary Figure 2), perhaps because plants and litter are absent in the barren arid soils tested here, and therefore further research is required. However, under light rain, higher temperature or diurnal cycles no clear trends were apparent, indicating a mutual effect of rain intensity and lower temperature on the patterns of bacterial ribosomal abundance.

The community composition changed only under heavy rain and the lower ambient temperature (Figure 5). Further analysis suggested that water content and salinity (measured by EC) were significant variables changing the community composition (Supplementary Figure 2). This change entailed an increase of Firmicutes and Bacteroidetes populations and a decrease in Actinobacteria and Verrucomicrobia (Figure 5), akin to previous reports testing hydration of soil crust (Angel and Conrad, 2013), arid (Št'ovíček et al., 2017), and semi-arid (Cruz-Martinez et al., 2012; Placella et al., 2012) soils. The Actinobacteria taxa which dominate the dry arid soil, was previously shown to have lineage-specific response to soil moisture (Cruz-Martinez et al., 2012) and to decrease upon hydration (Angel and Conrad, 2013) regaining dominance with desiccation (Št'ovíček et al., 2017).

The soil water content increased and decreased similarly during the first 72 h after the simulated heavy rain events, regardless of the incubation temperature (Figure 2B). However, the bacterial community changed only under the lower temperature. We thus speculate that rainfall-mediated changes in arid soil bacterial communities may occur only under heavy rain and lower temperatures (Figure 2). This could be attributed to the bacterial community's “reluctance” to change when sensing high temperatures, because these would result in rapid desiccation. This strategy has been proposed for desert plants, which avoid germination after a rain event when the future is uncertain. Experiments have shown that hydration of seeds under high temperature inhibits the germination of annuals and perennials (Khan and Ungar, 1996; Facelli et al., 2005). This was justified by suggesting that high temperatures signal that a given rain event is not likely to repeat and that the soil moisture will desiccate rapidly. During the winter in the desert, temperatures are lower, evaporation decreases, and the likelihood of hydration events increases. Therefore, when a heavy rain event occurs, there is a higher likelihood that the soil moisture will persist long enough to allow successful germination. The presented hypothesis that members of soil bacterial communities might also gauge desert rain events and ‘decide’ whether to proliferate or persist is intriguing, but requires further intense study under controlled and field conditions.

## Author contributions

OG and MS developed the concept and designed the experiments; AA and MS performed the experiments; AS analyzed the data; AS, OG, and MS wrote and edited the manuscript.

### Conflict of interest statement

The authors declare that the research was conducted in the absence of any commercial or financial relationships that could be construed as a potential conflict of interest.
