# Supplementary material for: The Impact of Hydration and Temperature on Bacterial Diversity in Arid Soil Mesocosms
Source: Front Microbiol. 2017 Jun 14;8:1078. doi: 10.3389/fmicb.2017.01078 (PMC5469873; doi:10.3389/fmicb.2017.01078)
Supplement: Supplementary file 1 [file DataSheet1.pdf]

Supplementary Tables

Supplementary Table 1. 16S rRNA primers used in this study**.**

**Target**	**Primer**	**Sequence (5'→3')**	**Source**
Total bacteria	S-D-Bact-0341-a-S-17	CCTACGGGAGGCAGCA	Klindworth et al., 2012
	S-*-Bact-0515-a-S-19	TTACCGCGGCTGCTGGCAC	
Actinobacteria	S-C-Act-235-a-20	CGCGGCCTATCAGCTTGTTG	Stach et al., 2003
	S-Bact-0515-a-S-19	TTACCGCGGCTGCTGGCAC	Klindworth et al., 2012
Firmicutes	928F-FirmF	TGAAACTYAAAGGAATTGACG	Bacchetti de Gregoris et al., 2011
	1014FFirmR	ACCATGCAACCACCTGTC	

References

Bacchetti De Gregoris, T., Aldred, N., Clare, A. S., and Burgess, J. G. (2011). Improvement of phylum- and class-specific primers for real-time PCR quantification of bacterial taxa. *J. Microbiol. Methods* 86, 351–6. doi:Research Support, Non-U.S. Gov't.

Klindworth, A., Pruesse, E., Schweer, T., Peplies, J., Quast, C., Horn, M., et al. (2012). Evaluation of general 16S ribosomal RNA gene PCR primers for classical and next-generation sequencing-based diversity studies. *Nucleic Acids Res.* 41, e1–e1. doi:10.1093/nar/gks808.

Stahl, D. A., and Amman, R. (1991). "Development and application of nucleic acid probes in bacterial systematics," in *Nucleic acid techniques in bacterial systematics*, eds. E. Stackebrandt and M. Goodfellow (Chichester, England: John Wiley & Sons Ltd), 205–248.

Supplementary Table 2. Soil physico-chemical composition throughout the experiment.

Time (days)	EC		pH		Ammonium		Nitrite		Nitrate	
Units	ds/m				mg/kg soil		mg/kg soil		mg/kg soil	
**25 ºC, 50 mm**										
	Ave	STD	Ave	STD	Ave	STD	Ave	STD	Ave	STD
0.0	1.91	NA	7.90	NA						
0.5	1.85	0.02	8.06	0.03	7.8	0.0	0.07	0.00	5.5	0.0
1.5	1.49	0.02	8.06	0.01	9.1	0.4	0.36	0.03	3.3	0.4
3.0	1.73	0.03	8.06	0.01	6.2	0.2	0.31	0.03	2.5	0.3
7.0	1.62	0.03	8.26	0.03	9.4	0.2	-0.02	0.02	2.9	0.2
14.0	1.36	0.04	8.30	0.05	8.3	0.6	0.19	0.00	4.3	0.2
21.0	1.33	0.03	8.08	0.05	3.3	0.3	0.17	0.00	6.9	0.6
**25 ºC, 10 mm**										
0.0	1.20	0.01	8.20	0.20	3.6	1.2	0.03	0.01	4.0	0.4
0.5	1.21	0.04	8.42	0.02	11.2	0.3	0.51	0.03	2.8	0.3
1.5	1.18	0.04	8.44	0.02	8.9	0.3	0.44	0.02	2.8	0.3
3.0	1.06	0.02	8.42	0.02	8.8	0.2	0.37	0.03	3.1	0.4
7.0	1.13	0.02	8.31	0.02	6.2	0.1	0.34	0.04	5.4	0.4
14.0	1.10	0.02	8.50	0.01	6.1	0.1	0.48	0.02	5.9	0.4
21.0	1.49	0.02	8.16	0.04	9.6	0.5	0.27	0.01	6.9	0.5
**36 ºC, 50 mm**										
0.0	1.19	0.02	8.85	0.09	41.0	4.9	NA	NA	4.2	0.0
0.5	1.19	0.02	8.98	0.02	41.6	3.9	0.64	0.03	3.0	0.2
1.5	1.09	0.01	8.70	0.01	16.6	1.2	0.43	0.02	3.8	0.2
3.0	1.03	0.01	8.56	0.03	10.7	0.3	0.30	0.01	4.6	0.2
7.0	1.11	0.02	8.38	0.01	6.7	0.1	0.28	0.01	6.4	0.3
14.0	1.49	0.03	8.51	0.04	21.1	1.8	0.28	0.01	3.6	0.4
21.0	1.57	0.05	8.45	0.03	44.0	4.9	0.29	0.01	3.0	0.4

36/10 ºC, 50 mm										
0.0	NA	NA	7.90	0.06	51.8	4.1	0.31	0.03	7.0	0.2
0.5	NA	NA	8.05	0.04	41.9	2.0	0.47	0.01	2.8	0.3
1.5	NA	NA	8.11	0.02	18.1	0.6	0.38	0.03	3.0	0.3
3.0	1.16	0.02	7.82	0.04	9.0	0.5	0.42	0.03	2.5	0.2
7.0	1.24	0.03	8.04	0.03	8.5	0.2	0.51	0.01	5.5	0.6
14.0	1.45	0.06	7.84	0.02	11.0	0.7	0.38	0.03	2.8	0.3
21.0	1.32	0.03	8.41	0.02	11.5	0.3	0.43	0.02	12.7	0.8

Supplementary Table 3. Bacterial community composition throughout the experiments. Taxonomic data is presented on the class level. Each time point is a sum of three replicate points. Time zero is a sum of all four sampling times and hence identical for all experiments. Data is taken from evenly subsampled (rarefied) dataset, but is not normalized. Classes that accounted for more than 500 individuals throughout the whole dataset were removed.

Time (days)	Acido bacteria	Actino bacteria	Armatim onadetes	Bacteroi detes	Chloro flexi	Cyano bacteria	Firmi cutes	Gemmai monades	Plancto mycetes	Proteo bacteria	Thauma rchaeota	Verruco microbia	Unclass ified
**25°C, 50 mm**													
0	3282	457266	126	9610	5250	2044	22638	1335	13350	196086	1155	15756	194
0.5	1932	120870	32	44000	1752	44	184536	1390	4280	299640	180	3152	81
1.5	2064	109344	54	42440	2208	288	187880	2220	4780	172524	138	6448	26
7	2196	155040	88	40420	1788	40	206624	1760	4620	180114	108	2912	54
14	1656	126582	12	39000	1452	44	230340	1040	3860	201894	246	2472	36
21	1560	177276	130	38180	2100	56	179960	670	4780	218196	210	5200	73
**25°C, 10 mm**													
0	3282	457266	126	9610	5250	2044	22638	1335	13350	196086	1155	15756	194
0.5	4740	531624	206	5620	7236	180	17908	3710	16400	168696	1062	13000	100
1.5	5028	415038	152	9700	5124	3032	26004	2520	14680	186912	1074	14936	98
7	3960	505206	348	12600	7200	124	49456	2390	16700	144474	780	11816	110
14	3444	529380	182	5560	6312	1744	12540	1580	16060	201432	1134	13728	125
21	4044	413916	102	4440	5772	4640	3388	690	16320	233772	972	19176	116

**36°C, 50 mm**													
0	3282	457266	126	9610	5250	2044	22638	1335	13350	196086	1155	15756	194
0.5	3636	376278	72	7480	6540	4232	10560	3260	12180	226182	930	14192	119
1.5	3300	437376	76	11880	5268	3096	11748	3980	9040	212124	1404	8048	150
7	4896	424320	92	11960	7596	1484	22440	4610	14680	190080	1014	9776	157
14	2904	508062	78	11560	7044	432	38368	2620	15620	189618	912	9144	115
21	2964	583032	128	3340	4404	1092	1144	1480	13700	247698	1206	11112	114
**36/10°C, 50 mm**													
0	3282	457266	126	9610	5250	2044	22638	1335	13350	196086	1155	15756	194
0.5	2424	667692	124	5300	10860	524	20108	1880	16260	157674	780	9592	106
1.5	2868	590580	108	6620	9576	352	26048	3600	15080	166716	1476	4992	163
3	2856	547944	120	10100	9492	208	33308	5410	16760	178332	828	4056	146
7	2952	449208	122	6720	5760	1884	16588	2830	8780	255156	912	12024	117
14	708	119391	23	2550	2364	166	9328	330	3300	51414	183	3336	60
21	2484	583950	106	11700	7572	116	48752	1610	14680	185922	720	6272	77

Supplementary Figure

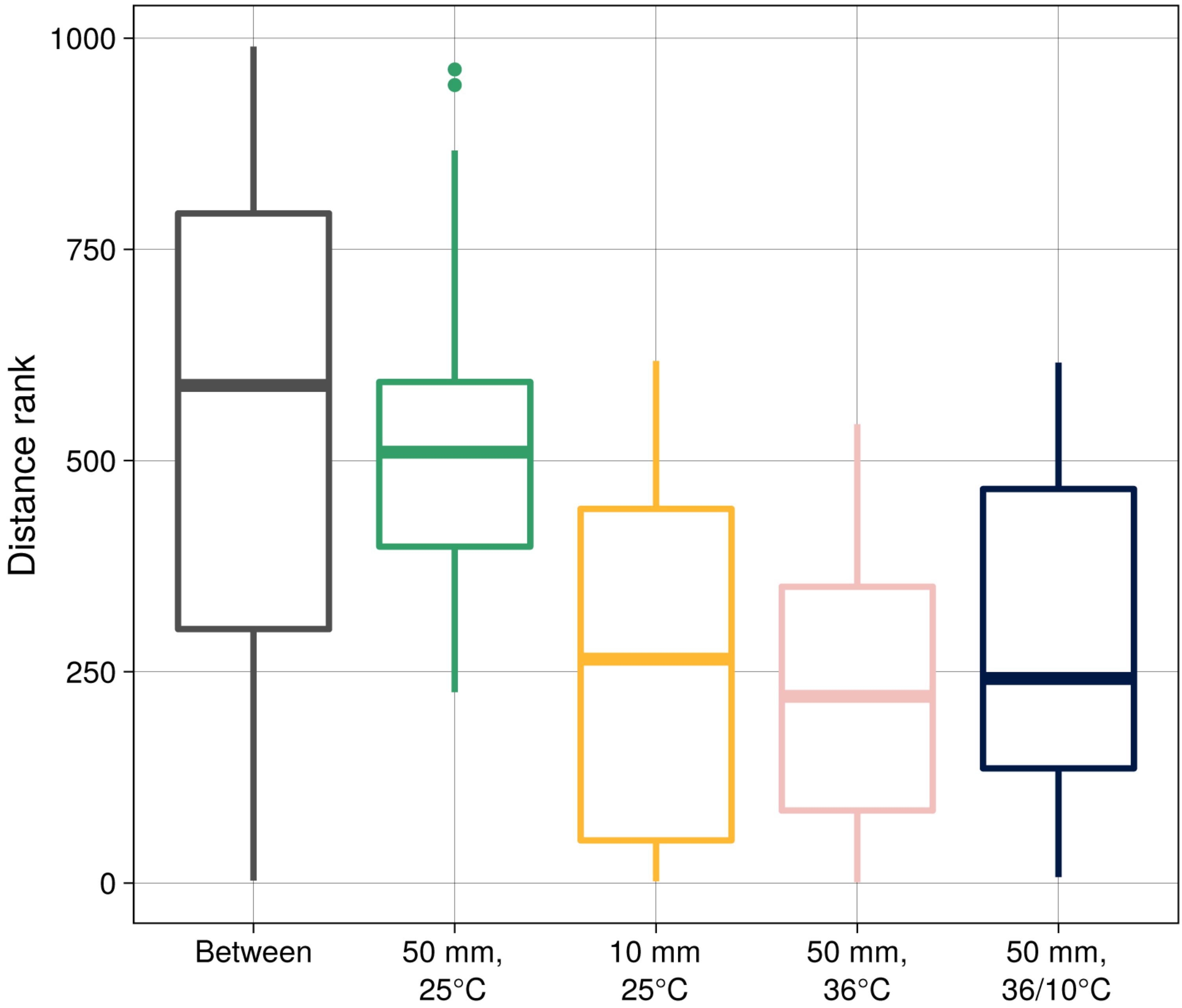

SUPPLEMENTARY FIGURE 1 Graphical representation of the results of the ANOSIM analysis of the groups represented in Figure 5. The null hypothesis is rejected and therefore the clusters are not due to random distribution (R = 0.3797, p < 2.10-5, 50,000 permutations).

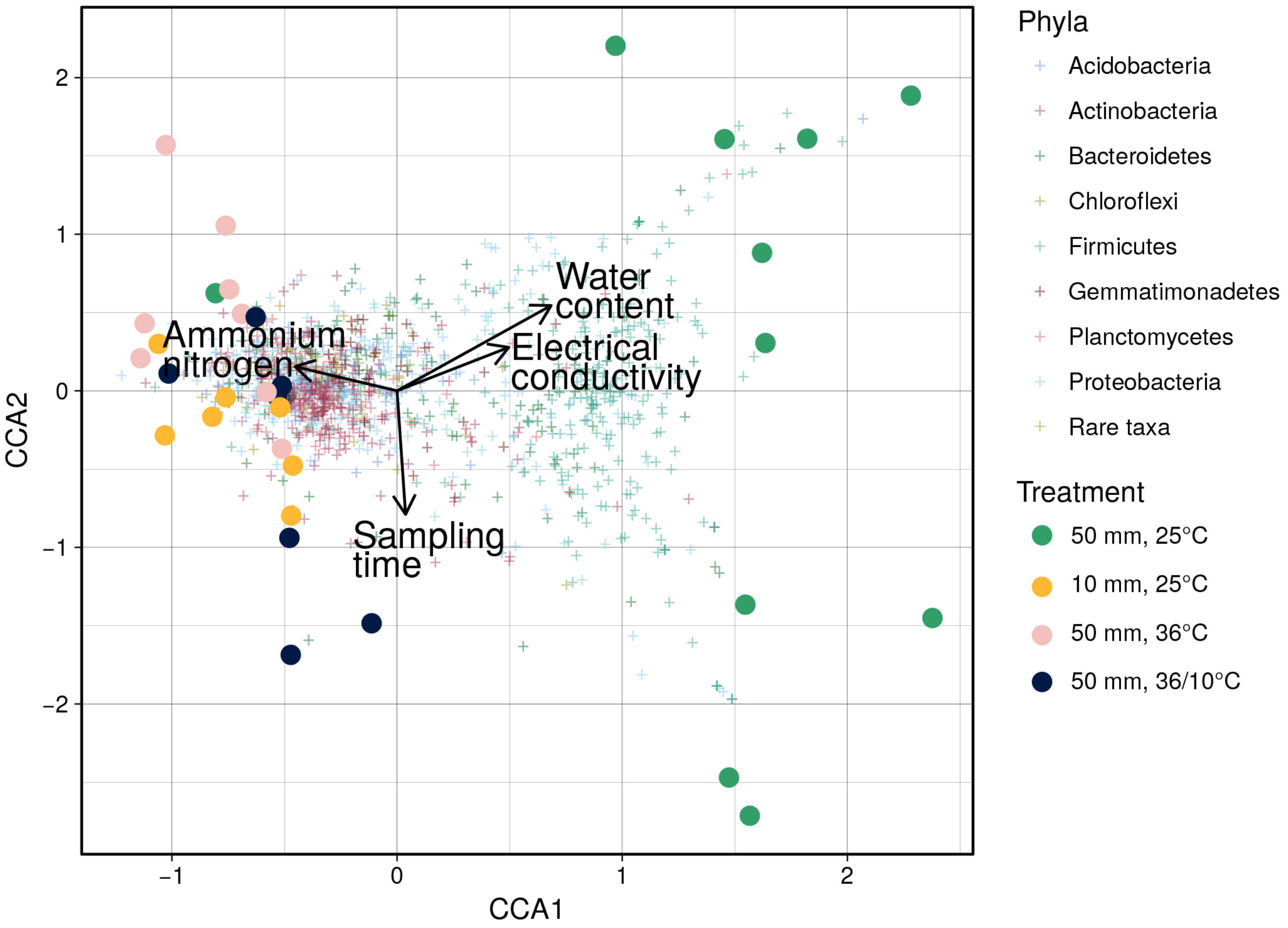

SUPPLEMENTARY FIGURE 2. Canonical Correspondence Analysis (CCA) compares soil bacterial communities (dots and crosses) and physico-chemical parameters (arrows). Colors indicate heavy rain and mild temperature (green dots), light rain and mild temperatures (yellow dots), heavy rain and high temperature (pink dots) and heavy rain and temperature diurnal cycle (black dots). Arrows indicate the direction and magnitude by measurable variables associated with bacteria community structures. The first two axes represent the relationships between environmental variables and bacterial diversity at the phyla level. CCA1 captures 26.8% of the variance in the microbial communities' data and is mostly influenced by water content and electrical conductivity ($p<0.002$). CCA2 captures 2.7% of the microbial variance and is mostly influence by sampling time ($p<0.004$).

Supplementary File 1

Generalized linear mixed-effects model: Eveness

Loading dataset

Replicate 1 and 2 from each dataset were loaded for a comparison between the dataset of unequal sizes. The dataset consists of soil chemical properties such as electric conductivity, pH, water content, ammonium, nitrite and nitrate as explanatory variables and the species richness as a response variable.

Null model

The null model was constructed to verify that the dataset contains true replicates that therefore don't need to be included in the model.

```
## summary from lme4 is returned
## some computational error has occurred in lmerTest
```

```
## [1] "Linear mixed model fit by REML ['lmerMod']"
## [2] "Formula: Pielou ~ 1 + (1 | Replicate)"
## [3] "   Data: structure(list(Name = structure(c(27L, 34L, 41L, 4L, 20L, 1L,  "
```

```
##  [1] ""
##  [2] "Scaled residuals: "
##  [3] "    Min      1Q  Median      3Q     Max "
##  [4] "-3.3460 -0.2568  0.2628  0.7047  1.1190 "
##  [5] ""
##  [6] "Random effects:"
##  [7] " Groups    Name        Variance Std.Dev."
##  [8] " Replicate (Intercept) 0.000000 0.00000 "
##  [9] " Residual              0.001301 0.03607 "
## [10] "Number of obs: 45, groups:  Replicate, 2"
## [11] ""
## [12] "Fixed effects:"
## [13] "            Estimate Std. Error t value"
## [14] "(Intercept) 0.590532   0.005378   109.8"
```

The replicates do not explain any variance and therefore will not be considered in the following models.

Model including the entire dataset

A model including soil chemical properties such as electric conductivity, pH, water content, ammonium, nitrite and nitrate as explanatory variables and the species richness as a response variable.

```
## Linear mixed model fit by REML t-tests use Satterthwaite approximations
##   to degrees of freedom [lmerMod]
## Formula: Pielou ~ EC + pH + N.NH4 + NO2 + NO3 + TimeValue + WC + (1 |
##     Column)
##    Data: df1
##
## REML criterion at convergence: -85.7
##
## Scaled residuals:
##      Min       1Q   Median       3Q      Max
## -2.59564 -0.50682 -0.03348  0.60233  1.48416
##
## Random effects:
##  Groups   Name        Variance  Std.Dev.
##  Column   (Intercept) 0.0005059 0.02249
##  Residual             0.0006301 0.02510
## Number of obs: 34, groups:  Column, 4
##
## Fixed effects:
##              Estimate Std. Error        df t value Pr(>|t|)
## (Intercept)  1.128791   0.293988 20.398000   3.840 0.000994 ***
## EC          -0.085757   0.033959 12.908000  -2.525 0.025460 *
## pH          -0.048135   0.034609 18.926000  -1.391 0.180411
## N.NH4        0.001423   0.001110 21.724000   1.282 0.213483
## NO2          0.033349   0.044898 21.838000   0.743 0.465539
## NO3          0.003426   0.002998 25.269000   1.143 0.263907
## TimeValue   -0.003257   0.001024 21.597000  -3.181 0.004394 **
## WC          -0.006060   0.001983 11.702000  -3.057 0.010227 *
## ---
## Signif. codes:  0 '***' 0.001 '**' 0.01 '*' 0.05 '.' 0.1 ' ' 1
##
## Correlation of Fixed Effects:
##           (Intr) EC     pH     N.NH4  NO2    NO3    TimeVl
## EC        -0.498
## pH        -0.986  0.375
## N.NH4      0.432 -0.656 -0.395
## NO2        0.068 -0.014 -0.089 -0.210
## NO3        0.470 -0.304 -0.506  0.616 -0.082
## TimeValue -0.118  0.421  0.048 -0.499  0.035 -0.430
## WC        -0.081  0.245 -0.011  0.154 -0.399  0.261  0.369
```

Model simplification

```
## refitting model(s) with ML (instead of REML)
```

```
## Data: df2
## Models:
## ..1: Pielou ~ TimeValue + WC + EC + (1 | Column)
## object: Pielou ~ pH + N.NH4 + NO2 + NO3 + TimeValue + WC + (1 | Column)
##        Df     AIC     BIC logLik deviance Chisq Chi Df Pr(>Chisq)
## ..1     6 -138.92 -129.76 75.461  -150.92
## object  9 -129.57 -115.83 73.784  -147.57     0      3          1
```

The null hypothesis can not be rejected and therefore it's assumed that the simplified model is not statistically different from the original model and therefore it will be retained.

Simplified model assumptions

Variance inflation factors and multicolinearity

```
## TimeValue        WC        EC
##  1.520555  1.652899  1.109005
```

The variance inflation factors of all the variables were found satisfactory.

Q-Q plot

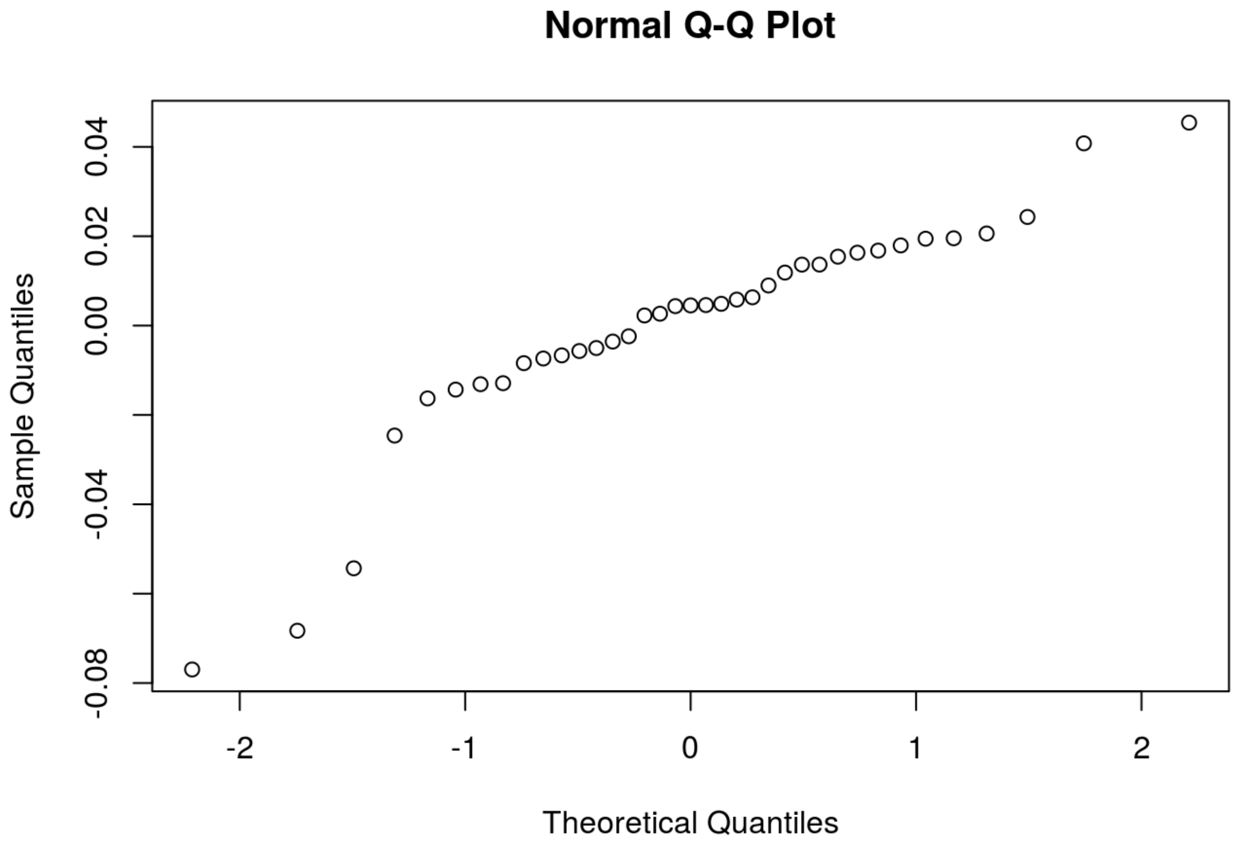

Final results

```
## Linear mixed model fit by REML t-tests use Satterthwaite approximations
##   to degrees of freedom [lmerMod]
## Formula: Pielou ~ TimeValue + WC + EC + (1 | Column)
##    Data: df1
##
## REML criterion at convergence: -120.8
##
## Scaled residuals:
##     Min      1Q  Median      3Q     Max
## -2.8338 -0.2714  0.1661  0.5678  1.6722
##
## Random effects:
##  Groups   Name        Variance  Std.Dev.
##  Column   (Intercept) 0.0005471 0.02339
##  Residual             0.0007377 0.02716
## Number of obs: 37, groups:  Column, 4
##
## Fixed effects:
##               Estimate Std. Error         df t value Pr(>|t|)
## (Intercept)  0.6594875  0.0406159 22.4800000  16.237 6.62e-14 ***
## TimeValue   -0.0013938  0.0007759 32.9600000  -1.796   0.0816 .
## WC          -0.0025343  0.0013737 31.4900000  -1.845   0.0745 .
## EC          -0.0297538  0.0248006 31.6300000  -1.200   0.2392
## ---
## Signif. codes:  0 '***' 0.001 '**' 0.01 '*' 0.05 '.' 0.1 ' ' 1
##
## Correlation of Fixed Effects:
##           (Intr) TimeVl WC
## TimeValue -0.382
## WC        -0.567  0.582
## EC        -0.891  0.119  0.305
```

Supplementary File 2

Generalized linear mixed-effects model: qPCR

Loading dataset

Since qPCR was measured in two technical replicates, an average of the two was calculated prior to the data comparison. The electric conductivity of the soil was not included in the dataset, since too many values in that dataset were missing.

Total bacteria ribosomal count

Null model

The null model was constructed to verify that the dataset contains true replicates that therefore don't need to be included in the model.

```
## summary from lme4 is returned
## some computational error has occurred in lmerTest
```

```
## Linear mixed model fit by REML ['lmerMod']
## Formula: log(TotalBacteria) ~ 1 + (1 | Replicate)
##    Data: df1
##
## REML criterion at convergence: 324
##
## Scaled residuals:
##     Min      1Q  Median      3Q     Max
## -2.3012 -0.5508  0.1534  0.4443  1.9427
##
## Random effects:
##  Groups    Name        Variance Std.Dev.
##  Replicate (Intercept) 0.000    0.000
##  Residual              4.157    2.039
## Number of obs: 76, groups:  Replicate, 3
##
## Fixed effects:
##             Estimate Std. Error t value
## (Intercept)  19.3724     0.2339   82.83
```

The replicates do not explain any variance and therefore will not be considered in the following models.

Model including the entire dataset

```
##
## Call:
## lm(formula = log(TotalBacteria) ~ Experiment + TimeValue + WC +
##     pH + N.NH4 + N.NO3 + N.NO2, data = df1)
##
## Residuals:
##     Min      1Q  Median      3Q     Max
## -2.7110 -0.9766 -0.1406  1.0210  3.3481
##
## Coefficients:
##                         Estimate Std. Error t value Pr(>|t|)
## (Intercept)              9.77549    5.47853   1.784 0.079693 .
## ExperimentExperiment 2   2.22308    0.84603   2.628 0.011027 *
## ExperimentExperiment 3   2.77242    0.68613   4.041 0.000161 ***
## ExperimentExperiment 4   1.50122    0.71202   2.108 0.039406 *
## TimeValue               -0.07354    0.03623  -2.030 0.047045 *
## WC                      -0.13297    0.06487  -2.050 0.045015 *
## pH                       1.06332    0.66504   1.599 0.115374
## N.NH4                    0.04507    0.01602   2.814 0.006707 **
## N.NO3                    0.08489    0.08808   0.964 0.339181
## N.NO2                   -1.03742    1.70226  -0.609 0.544658
## ---
## Signif. codes:  0 '***' 0.001 '**' 0.01 '*' 0.05 '.' 0.1 ' ' 1
##
## Residual standard error: 1.487 on 57 degrees of freedom
##   (17 observations deleted due to missingness)
## Multiple R-squared:  0.5573, Adjusted R-squared:  0.4874
## F-statistic: 7.973 on 9 and 57 DF,  p-value: 1.615e-07
```

Model simplification

```
## Analysis of Variance Table
##
## Model 1: log(TotalBacteria) ~ Experiment + TimeValue + WC + pH + N.NH4 +
##     N.NO3 + N.NO2
## Model 2: log(TotalBacteria) ~ Experiment + WC + N.NH4
##   Res.Df    RSS Df Sum of Sq      F Pr(>F)
## 1     34 47.943
## 2     38 53.208 -4   -5.2654 0.9335 0.4562
```

```
##
## Call:
## lm(formula = log(TotalBacteria) ~ Experiment + WC + N.NH4, data = df2)
##
## Residuals:
##     Min      1Q  Median      3Q     Max
## -1.9046 -0.6129 -0.0395  0.4235  3.3091
##
## Coefficients:
##                        Estimate Std. Error t value Pr(>|t|)
## (Intercept)            16.88895    0.54921  30.751  < 2e-16 ***
## ExperimentExperiment 2   3.04599    0.59570   5.113 9.31e-06 ***
## ExperimentExperiment 3   3.42549    0.56680   6.044 4.96e-07 ***
## ExperimentExperiment 4   1.74989    0.55942   3.128  0.00337 **
## WC                     -0.10096    0.04052  -2.492  0.01718 *
## N.NH4                   0.07069    0.01490   4.743 2.95e-05 ***
## ---
## Signif. codes:  0 '***' 0.001 '**' 0.01 '*' 0.05 '.' 0.1 ' ' 1
##
## Residual standard error: 1.183 on 38 degrees of freedom
## Multiple R-squared:  0.7882, Adjusted R-squared:  0.7603
## F-statistic: 28.28 on 5 and 38 DF,  p-value: 7.642e-12
```

```
## Linear mixed model fit by REML t-tests use Satterthwaite approximations
##   to degrees of freedom [lmerMod]
## Formula: log(TotalBacteria) ~ N.NH4 + WC + (1 | Experiment)
##    Data: df1
##
## REML criterion at convergence: 281
##
## Scaled residuals:
##     Min      1Q  Median      3Q     Max
## -1.8132 -0.6985 -0.1009  0.7163  2.5675
##
## Random effects:
##  Groups     Name        Variance Std.Dev.
##  Experiment (Intercept) 0.9739   0.9869
##  Residual               2.1853   1.4783
## Number of obs: 73, groups:  Experiment, 4
##
## Fixed effects:
##             Estimate Std. Error       df t value Pr(>|t|)
## (Intercept) 19.17112    0.62988  5.55000  30.436 2.17e-07 ***
## N.NH4        0.05024    0.01390 69.47000   3.615 0.000565 ***
## WC          -0.08040    0.03763 69.98000  -2.137 0.036106 *
## ---
## Signif. codes:  0 '***' 0.001 '**' 0.01 '*' 0.05 '.' 0.1 ' ' 1
##
## Correlation of Fixed Effects:
##       (Intr) N.NH4
## N.NH4 -0.361
## WC    -0.430  0.015
```

The null hypothesis can not be rejected and therefore it's assumed that the simplified model is not statistically different from the original model and therefore it will be retained.

Simplified model assumptions

Variance inflation factors and multicolinearity

```
##                GVIF Df GVIF^(1/(2*Df))
## Experiment 1.976289  3        1.120233
## WC         1.356475  1        1.164678
## N.NH4      1.457991  1        1.207473
```

The variance inflation factors of all the variables were found satisfactory.

Q-Q plot

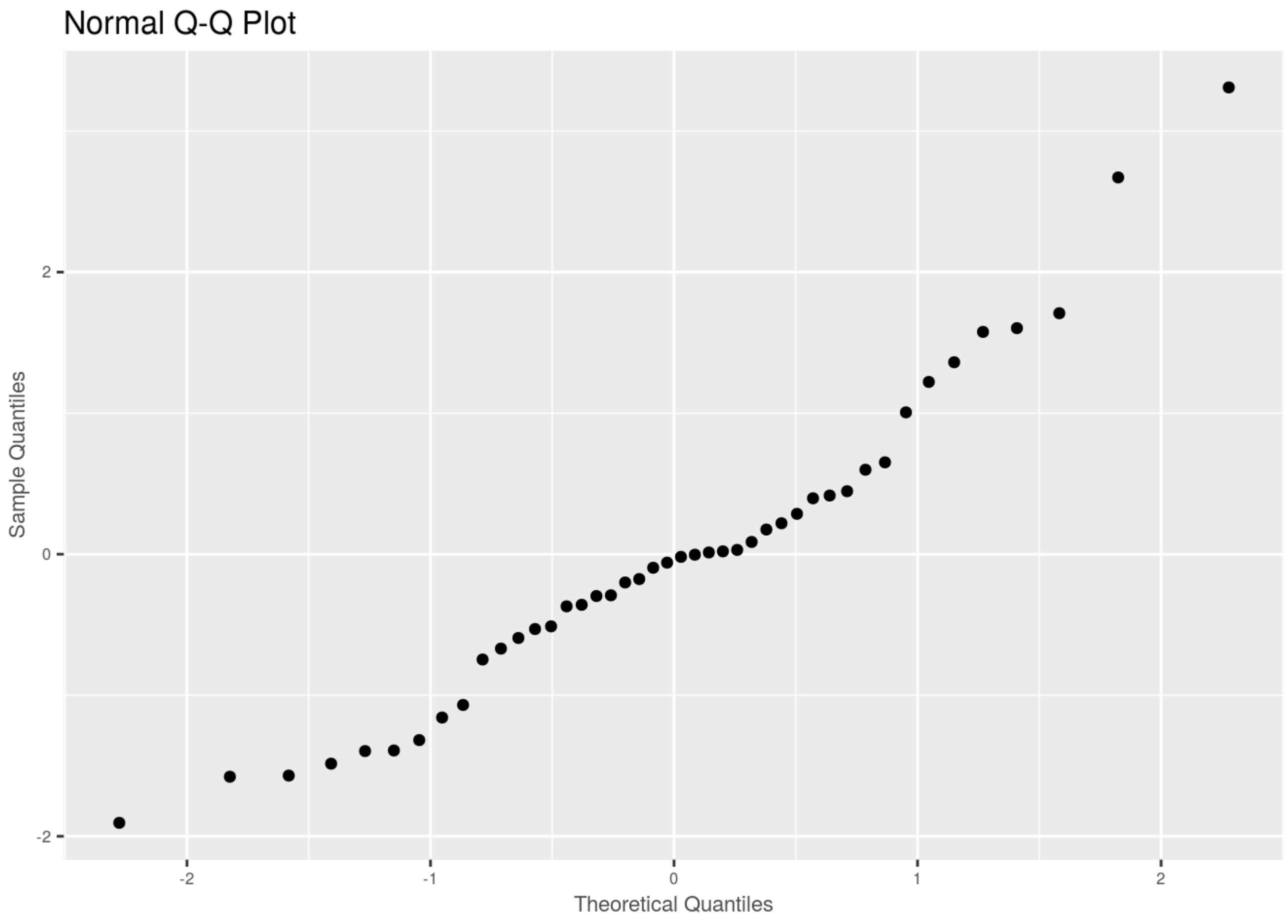

Final results

```
##
## Call:
## lm(formula = log(TotalBacteria) ~ Experiment + WC + N.NH4, data = df2)
##
## Residuals:
##     Min      1Q  Median      3Q     Max
## -1.9046 -0.6129 -0.0395  0.4235  3.3091
##
## Coefficients:
##                        Estimate Std. Error t value Pr(>|t|)
## (Intercept)            16.88895    0.54921  30.751  < 2e-16 ***
## ExperimentExperiment 2  3.04599    0.59570   5.113 9.31e-06 ***
## ExperimentExperiment 3  3.42549    0.56680   6.044 4.96e-07 ***
## ExperimentExperiment 4  1.74989    0.55942   3.128  0.00337 **
## WC                     -0.10096    0.04052  -2.492  0.01718 *
## N.NH4                   0.07069    0.01490   4.743 2.95e-05 ***
## ---
## Signif. codes:  0 '***' 0.001 '**' 0.01 '*' 0.05 '.' 0.1 ' ' 1
##
## Residual standard error: 1.183 on 38 degrees of freedom
## Multiple R-squared:  0.7882, Adjusted R-squared:  0.7603
## F-statistic: 28.28 on 5 and 38 DF,  p-value: 7.642e-12
```

Actinobacteria ribosomal count

Null model

The null model was constructed to verify that the dataset contains true replicates that therefore don't need to be included in the model.

```
## summary from lme4 is returned
## some computational error has occurred in lmerTest
```

```
## Linear mixed model fit by REML ['lmerMod']
## Formula: log(Actinobacteria) ~ 1 + (1 | Replicate)
##    Data: df1
##
## REML criterion at convergence: 347.6
##
## Scaled residuals:
##      Min       1Q   Median       3Q      Max
## -2.11303 -0.50567  0.08442  0.58632  2.10755
##
## Random effects:
##  Groups    Name        Variance Std.Dev.
##  Replicate (Intercept) 0.000    0.000
##  Residual              5.689    2.385
## Number of obs: 76, groups:  Replicate, 3
##
## Fixed effects:
##             Estimate Std. Error t value
## (Intercept)  16.9754     0.2736   62.04
```

The replicates do not explain any variance and therefore will not be considered in the following models.

Model including the entire dataset

```
## Linear mixed model fit by REML t-tests use Satterthwaite approximations
##   to degrees of freedom [lmerMod]
## Formula: log(Actinobacteria) ~ pH + N.NH4 + N.NO2 + N.NO3 + TimeValue +
##     WC + (1 | Experiment)
##    Data: df1
##
## REML criterion at convergence: 303.5
##
## Scaled residuals:
##     Min      1Q  Median      3Q     Max
## -2.1291 -0.6791 -0.1642  0.8427  2.3084
##
## Random effects:
##  Groups     Name        Variance Std.Dev.
##  Experiment (Intercept) 1.601    1.265
##  Residual               4.640    2.154
## Number of obs: 67, groups:  Experiment, 4
##
## Fixed effects:
##             Estimate Std. Error       df t value Pr(>|t|)
## (Intercept) 11.91492    7.89864 57.75000   1.508   0.1369
## pH           0.70516    0.96197 56.88000   0.733   0.4665
## N.NH4        0.03976    0.02250 59.71000   1.767   0.0823 .
## N.NO2       -1.53937    2.29422 50.08000  -0.671   0.5053
## N.NO3        0.01977    0.12673 58.09000   0.156   0.8766
## TimeValue   -0.04914    0.05121 59.98000  -0.959   0.3412
## WC          -0.08088    0.08615 44.51000  -0.939   0.3529
## ---
## Signif. codes:  0 '***' 0.001 '**' 0.01 '*' 0.05 '.' 0.1 ' ' 1
##
## Correlation of Fixed Effects:
##           (Intr) pH     N.NH4  N.NO2  N.NO3  TimeVl
## pH        -0.985
## N.NH4     -0.029 -0.019
## N.NO2     -0.075  0.020 -0.175
## N.NO3     -0.103  0.034  0.142 -0.080
## TimeValue  0.077 -0.145  0.077 -0.024 -0.345
## WC         0.145 -0.238  0.084 -0.329  0.173  0.537
```

Model simplification

```
## refitting model(s) with ML (instead of REML)
```

```
## Data: df2
## Models:
## ..1: log(Actinobacteria) ~ N.NH4 + WC + (1 | Experiment)
## object: log(Actinobacteria) ~ pH + N.NH4 + N.NO2 + N.NO3 + TimeValue +
## object:     WC + (1 | Experiment)
##        Df    AIC    BIC  logLik deviance Chisq Chi Df Pr(>Chisq)
## ..1     5 195.61 204.53 -92.806   185.61
## object  9 200.83 216.89 -91.417   182.83 2.779      4     0.5955
```

```
## Linear mixed model fit by REML t-tests use Satterthwaite approximations
##   to degrees of freedom [lmerMod]
## Formula: log(Actinobacteria) ~ N.NH4 + WC + (1 | Experiment)
##    Data: df2
##
## REML criterion at convergence: 193.8
##
## Scaled residuals:
##     Min      1Q  Median      3Q     Max
## -1.6102 -0.6056 -0.2051  0.4796  2.2877
##
## Random effects:
##  Groups     Name        Variance Std.Dev.
##  Experiment (Intercept) 2.296    1.515
##  Residual               3.541    1.882
## Number of obs: 44, groups:  Experiment, 4
##
## Fixed effects:
##             Estimate Std. Error       df t value Pr(>|t|)
## (Intercept) 16.48399    0.99121  5.97000  16.630 3.18e-06 ***
## N.NH4        0.06357    0.02307 40.78000   2.755  0.00873 **
## WC          -0.09353    0.06302 40.51000  -1.484  0.14550
## ---
## Signif. codes:  0 '***' 0.001 '**' 0.01 '*' 0.05 '.' 0.1 ' ' 1
##
## Correlation of Fixed Effects:
##       (Intr) N.NH4
## N.NH4 -0.344
## WC    -0.454 -0.025
```

```
## Linear mixed model fit by REML t-tests use Satterthwaite approximations
##   to degrees of freedom [lmerMod]
## Formula: log(Actinobacteria) ~ N.NH4 + WC + (1 | Experiment)
##    Data: df1
##
## REML criterion at convergence: 325.3
##
## Scaled residuals:
##     Min      1Q  Median      3Q     Max
## -2.4231 -0.7051 -0.1485  0.7624  2.2165
##
## Random effects:
##  Groups     Name        Variance Std.Dev.
##  Experiment (Intercept) 1.227    1.108
##  Residual               4.181    2.045
## Number of obs: 73, groups:  Experiment, 4
##
## Fixed effects:
##             Estimate Std. Error       df t value Pr(>|t|)
## (Intercept) 16.63714    0.77196  6.65000  21.552 2.09e-07 ***
## N.NH4        0.03988    0.01898 66.98000   2.101   0.0394 *
## WC          -0.03995    0.05162 69.68000  -0.774   0.4416
## ---
## Signif. codes:  0 '***' 0.001 '**' 0.01 '*' 0.05 '.' 0.1 ' ' 1
##
## Correlation of Fixed Effects:
##       (Intr) N.NH4
## N.NH4 -0.403
## WC    -0.482  0.015
```

The null hypothesis can not be rejected and therefore it’s assumed that the simplified model is not statistically different from the original model and therefore it will be retained.

Simplified model assumptions

Variance inflation factors and multicolinearity

```
##   N.NH4      WC
## 1.00024 1.00024
```

The variance inflation factors of all the variables were found satisfactory.

Q-Q plot

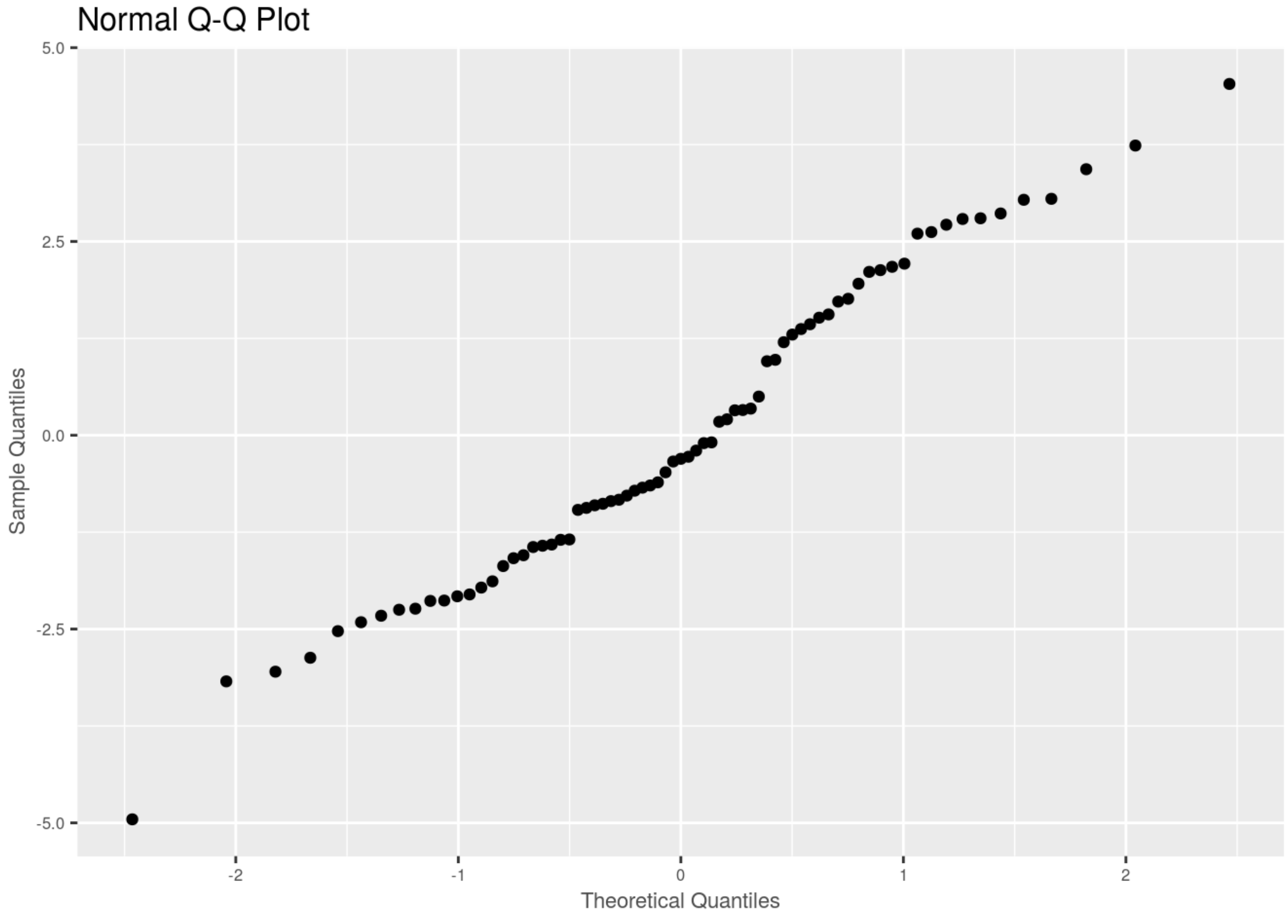

Final results

```
## Linear mixed model fit by REML t-tests use Satterthwaite approximations
##   to degrees of freedom [lmerMod]
## Formula: log(Actinobacteria) ~ N.NH4 + WC + (1 | Experiment)
##    Data: df1
##
## REML criterion at convergence: 325.3
##
## Scaled residuals:
##     Min      1Q  Median      3Q     Max
## -2.4231 -0.7051 -0.1485  0.7624  2.2165
##
## Random effects:
##  Groups     Name        Variance Std.Dev.
##  Experiment (Intercept) 1.227    1.108
##  Residual               4.181    2.045
## Number of obs: 73, groups:  Experiment, 4
##
## Fixed effects:
##             Estimate Std. Error       df t value Pr(>|t|)
## (Intercept) 16.63714    0.77196  6.65000  21.552 2.09e-07 ***
## N.NH4        0.03988    0.01898 66.98000   2.101   0.0394 *
## WC          -0.03995    0.05162 69.68000  -0.774   0.4416
## ---
## Signif. codes:  0 '***' 0.001 '**' 0.01 '*' 0.05 '.' 0.1 ' ' 1
##
## Correlation of Fixed Effects:
##       (Intr) N.NH4
## N.NH4 -0.403
## WC    -0.482  0.015
```

Firmicutes ribosomal count

Null model

The null model was constructed to verify that the dataset contains true replicates that therefore don’t need to be included in the model.

```
## summary from lme4 is returned
## some computational error has occurred in lmerTest
```

```
## Linear mixed model fit by REML ['lmerMod']
## Formula: log(Firmicutes) ~ 1 + (1 | Replicate)
##    Data: df1
##
## REML criterion at convergence: 215.6
##
## Scaled residuals:
##      Min       1Q   Median       3Q      Max
## -2.16971 -0.64020  0.05971  0.30588  2.42961
##
## Random effects:
##  Groups    Name        Variance Std.Dev.
##  Replicate (Intercept) 0.000    0.000
##  Residual              3.175    1.782
## Number of obs: 54, groups:  Replicate, 3
##
## Fixed effects:
##             Estimate Std. Error t value
## (Intercept)  13.8721     0.2425    57.2
```

The replicates do not explain any variance and therefore will not be considered in the following models.

Model including the entire dataset

```
## Linear mixed model fit by REML t-tests use Satterthwaite approximations
##   to degrees of freedom [lmerMod]
## Formula: log(Firmicutes) ~ pH + N.NH4 + N.NO2 + N.NO3 + TimeValue + WC +
##     (1 | Experiment)
##    Data: df1
##
## REML criterion at convergence: 161.9
##
## Scaled residuals:
##      Min       1Q   Median       3Q      Max
## -1.76313 -0.41545 -0.06206  0.38911  2.31769
##
## Random effects:
##  Groups     Name        Variance Std.Dev.
##  Experiment (Intercept) 2.207    1.486
##  Residual               1.413    1.189
## Number of obs: 45, groups:  Experiment, 4
##
## Fixed effects:
##             Estimate Std. Error       df t value Pr(>|t|)
## (Intercept) 20.09054    6.32197 36.32000   3.178  0.00303 **
## pH          -0.69510    0.75809 35.37000  -0.917  0.36540
## N.NH4        0.02863    0.01506 36.42000   1.901  0.06528 .
## N.NO2       -0.82775    1.60762 37.68000  -0.515  0.60964
## N.NO3        0.02260    0.09367 35.27000   0.241  0.81072
## TimeValue   -0.03375    0.03148 35.97000  -1.072  0.29079
## WC          -0.05683    0.06101 37.89000  -0.932  0.35746
## ---
## Signif. codes:  0 '***' 0.001 '**' 0.01 '*' 0.05 '.' 0.1 ' ' 1
##
## Correlation of Fixed Effects:
##           (Intr) pH     N.NH4  N.NO2  N.NO3  TimeVl
## pH        -0.984
## N.NH4     -0.119  0.067
## N.NO2     -0.034 -0.004 -0.090
## N.NO3     -0.222  0.150  0.103 -0.088
## TimeValue  0.129 -0.186  0.176 -0.077 -0.267
## WC         0.163 -0.241  0.140 -0.437  0.260  0.484
```

Model simplification

```
## refitting model(s) with ML (instead of REML)
```

```
## Data: df2
## Models:
## ..1: log(Firmicutes) ~ N.NO3 + (1 | Experiment)
## object: log(Firmicutes) ~ pH + N.NH4 + N.NO2 + N.NO3 + TimeValue + WC +
## object:     (1 | Experiment)
##        Df    AIC    BIC  logLik deviance  Chisq Chi Df Pr(>Chisq)
## ..1     4 162.32 169.46 -77.162   154.32
## object  9 163.07 179.13 -72.534   145.07 9.2546      5    0.09933 .
## ---
## Signif. codes:  0 '***' 0.001 '**' 0.01 '*' 0.05 '.' 0.1 ' ' 1
```

```
## Linear mixed model fit by REML t-tests use Satterthwaite approximations
##   to degrees of freedom [lmerMod]
## Formula: log(Firmicutes) ~ N.NO3 + (1 | Experiment)
##    Data: df1
##
## REML criterion at convergence: 170.3
##
## Scaled residuals:
##      Min       1Q   Median       3Q      Max
## -1.63897 -0.59441 -0.08138  0.27242  2.50029
##
## Random effects:
##  Groups     Name        Variance Std.Dev.
##  Experiment (Intercept) 2.429    1.558
##  Residual               1.479    1.216
## Number of obs: 49, groups:  Experiment, 4
##
## Fixed effects:
##             Estimate Std. Error       df t value Pr(>|t|)
## (Intercept) 13.74390    0.87585  4.34000  15.692 5.48e-05 ***
## N.NO3        0.02433    0.07793 44.24000   0.312    0.756
## ---
## Signif. codes:  0 '***' 0.001 '**' 0.01 '*' 0.05 '.' 0.1 ' ' 1
##
## Correlation of Fixed Effects:
##       (Intr)
## N.NO3 -0.411
```

The null hypothesis can not be rejected and therefore it’s assumed that the simplified model is not statistically different from the original model and therefore it will be retained.

Q-Q plot

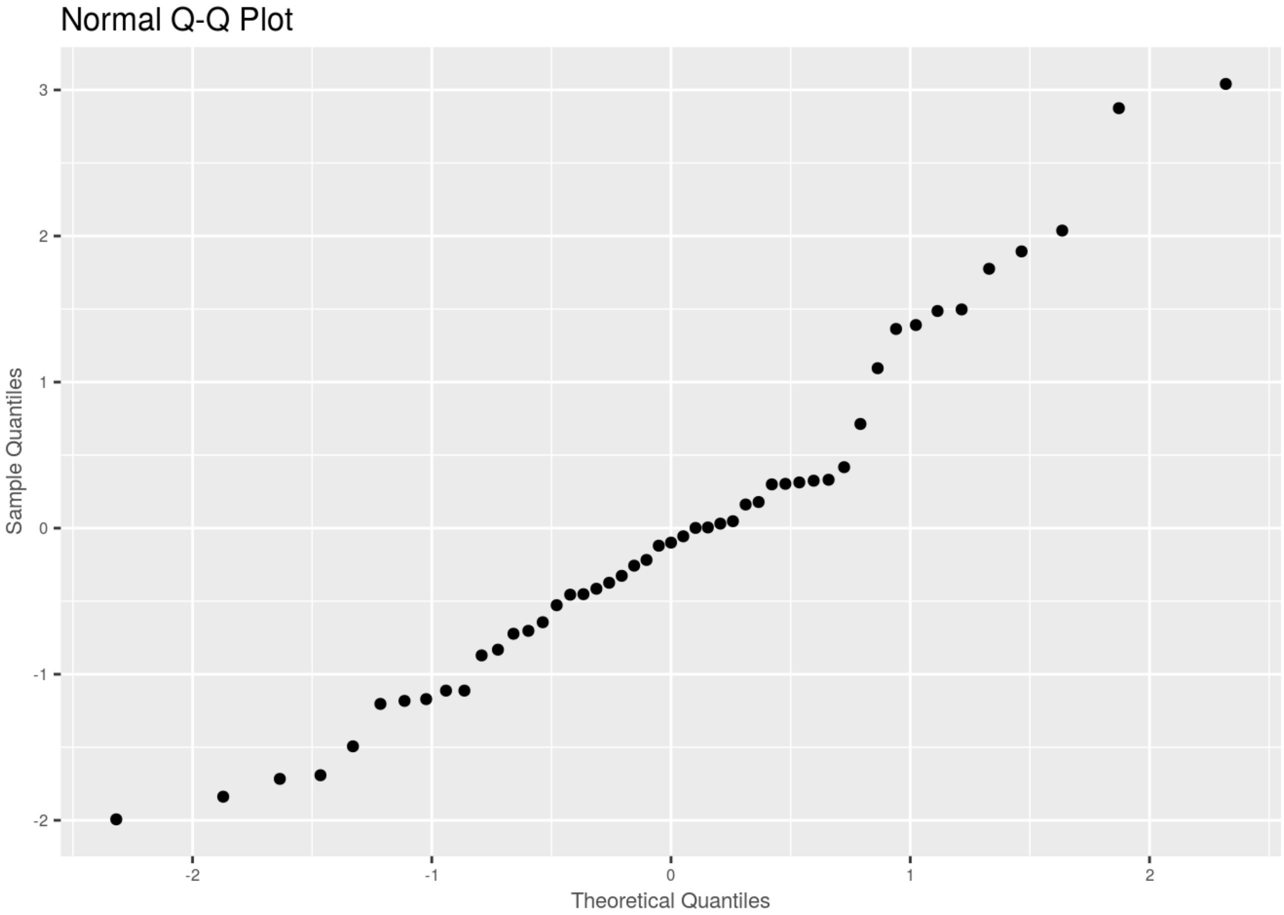

Final results

```
## Linear mixed model fit by REML t-tests use Satterthwaite approximations
##   to degrees of freedom [lmerMod]
## Formula: log(Firmicutes) ~ N.NO3 + (1 | Experiment)
##    Data: df1
##
## REML criterion at convergence: 170.3
##
## Scaled residuals:
##      Min       1Q   Median       3Q      Max
## -1.63897 -0.59441 -0.08138  0.27242  2.50029
##
## Random effects:
##  Groups     Name        Variance Std.Dev.
##  Experiment (Intercept) 2.429    1.558
##  Residual               1.479    1.216
## Number of obs: 49, groups:  Experiment, 4
##
## Fixed effects:
##             Estimate Std. Error       df t value Pr(>|t|)
## (Intercept) 13.74390    0.87585  4.34000  15.692 5.48e-05 ***
## N.NO3        0.02433    0.07793 44.24000   0.312    0.756
## ---
## Signif. codes:  0 '***' 0.001 '**' 0.01 '*' 0.05 '.' 0.1 ' ' 1
##
## Correlation of Fixed Effects:
##       (Intr)
## N.NO3 -0.411
```

Supplementary File 3

Generalized linear mixed-effects model: Richness

Loading dataset

Null model

The null model was constructed to verify that the dataset contains true replicates that therefore don't need to be included in the model.

```
## summary from lme4 is returned
## some computational error has occurred in lmerTest
```

```
## Linear mixed model fit by REML ['lmerMod']
## Formula: Species ~ 1 + (1 | Replicate)
##    Data: df1
##
## REML criterion at convergence: 570.8
##
## Scaled residuals:
##     Min      1Q  Median      3Q     Max
## -2.7112 -0.2641  0.4002  0.6634  1.1962
##
## Random effects:
##  Groups    Name        Variance Std.Dev.
##  Replicate (Intercept)     0      0
##  Residual              23110    152
## Number of obs: 45, groups:  Replicate, 2
##
## Fixed effects:
##             Estimate Std. Error t value
## (Intercept)   788.16      22.66   34.78
```

The replicates do not explain any variance and therefore will not be considered in the following models.

Model including the entire dataset

A model including soil chemical properties such as electric conductivity, pH, water content, ammonium, nitrite and nitrate as explanatory variables and the species richness as a response variable.

```
## Linear mixed model fit by REML t-tests use Satterthwaite approximations
##   to degrees of freedom [lmerMod]
## Formula: Species ~ EC + pH + N.NH4 + NO2 + NO3 + TimeValue + WC + (1 |
##     Column)
##    Data: df1
##
## REML criterion at convergence: 332.2
##
## Scaled residuals:
##      Min       1Q   Median       3Q      Max
## -2.00278 -0.51815  0.06899  0.62309  1.32466
##
## Random effects:
##  Groups   Name        Variance Std.Dev.
##  Column   (Intercept) 9670     98.34
##  Residual             5688     75.42
## Number of obs: 34, groups:  Column, 4
##
## Fixed effects:
##             Estimate Std. Error       df t value Pr(>|t|)
## (Intercept) 2809.782    935.864   25.359   3.002 0.005948 **
## EC          -288.872    112.625   19.886  -2.565 0.018527 *
## pH          -187.755    110.788   25.016  -1.695 0.102541
## N.NH4          4.533      3.513   25.670   1.290 0.208461
## NO2          268.268    142.466   25.361   1.883 0.071215 .
## NO3           10.156      9.305   25.938   1.091 0.285107
## TimeValue    -12.749      3.269   24.488  -3.900 0.000658 ***
## WC           -25.652      6.620   18.976  -3.875 0.001022 **
## ---
## Signif. codes:  0 '***' 0.001 '**' 0.01 '*' 0.05 '.' 0.1 ' ' 1
##
## Correlation of Fixed Effects:
##           (Intr) EC     pH     N.NH4  NO2    NO3    TimeVl
## EC        -0.471
## pH        -0.984  0.335
## N.NH4      0.420 -0.673 -0.372
## NO2        0.135 -0.109 -0.140 -0.155
## NO3        0.502 -0.291 -0.537  0.607 -0.046
## TimeValue -0.111  0.495  0.023 -0.517 -0.036 -0.385
## WC        -0.090  0.357 -0.019  0.077 -0.451  0.244  0.449
```

Model simplification

```
## refitting model(s) with ML (instead of REML)
```

```
## Data: df2
## Models:
## ..1: Species ~ EC + NO2 + TimeValue + WC + (1 | Column)
## object: Species ~ EC + pH + N.NH4 + NO2 + NO3 + TimeValue + WC + (1 |
## object:     Column)
##        Df    AIC   BIC  logLik deviance  Chisq Chi Df Pr(>Chisq)
## ..1     7 410.42 421.1 -198.21   396.42
## object 10 412.03 427.3 -196.02   392.03 4.3819      3     0.2231
```

The null hypothesis can not be rejected and therefore it’s assumed that the simplified model is not statistically different from the original model and therefore it will be retained.

Simplified model assumptions

Variance inflation factors and multicolinearity

```
##        EC       NO2 TimeValue        WC
##  1.436723  1.780937  2.244774  3.894904
```

The variance inflation factors of all the variables were found satisfactory.

Q-Q plot

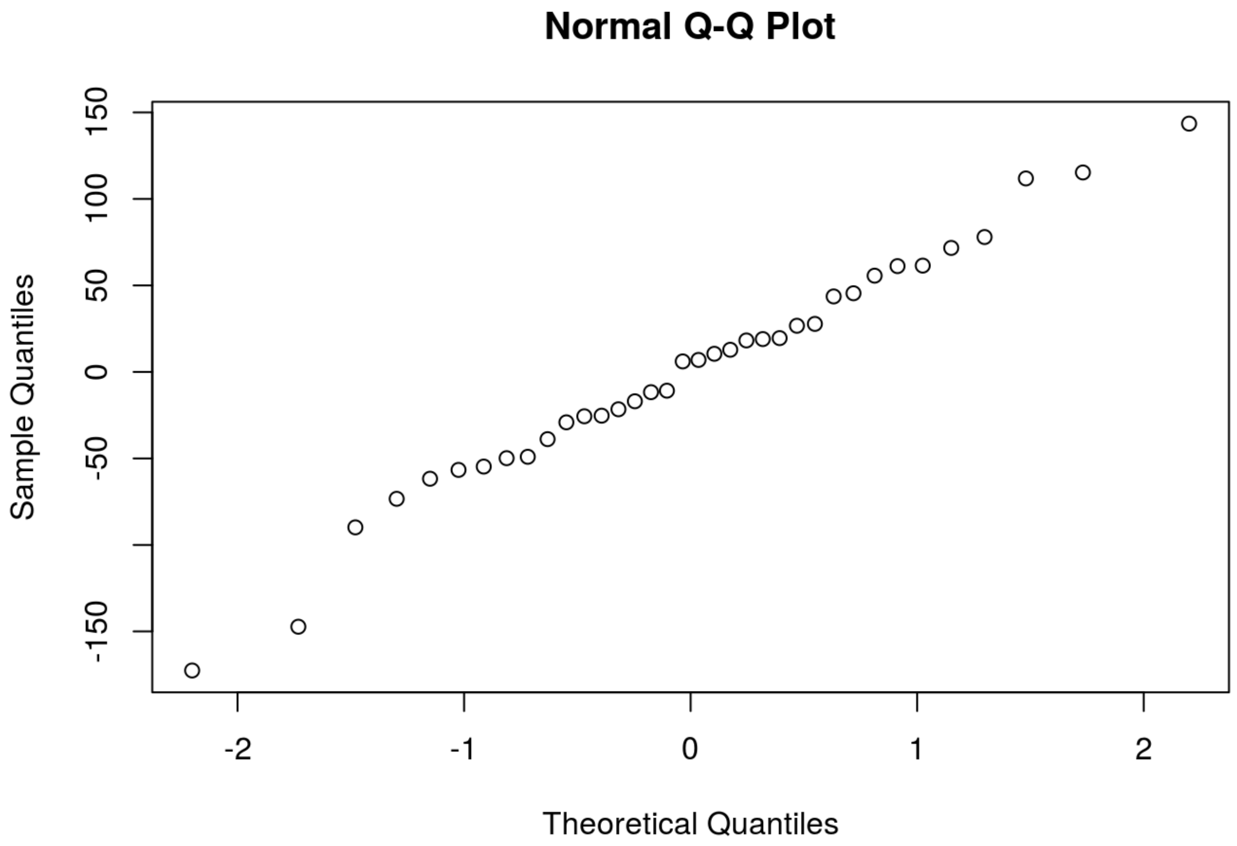
Normal Q-Q Plot
Sample Quantiles
150
100
50
0
-50
-150
-2
-1
0
1
2
Theoretical Quantiles

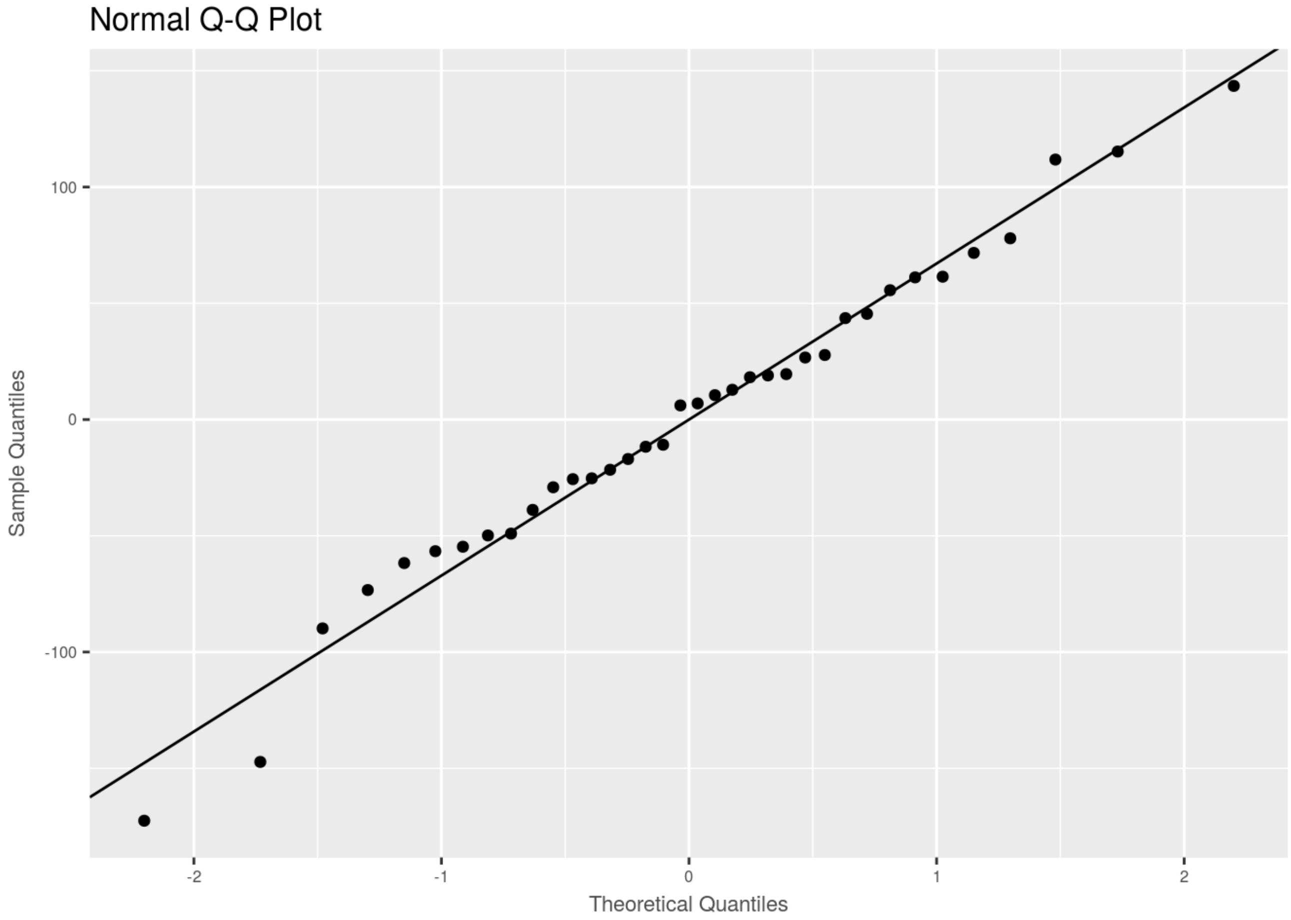
Normal Q-Q Plot
Sample Quantiles
100
0
-100
-2
-1
0
1
2
Theoretical Quantiles

Final results

```
## Linear mixed model fit by REML t-tests use Satterthwaite approximations
##   to degrees of freedom [lmerMod]
## Formula: Species ~ EC + NO2 + TimeValue + WC + (1 | Column)
##    Data: df1
##
## REML criterion at convergence: 380.3
##
## Scaled residuals:
##      Min       1Q   Median       3Q      Max
## -2.30251 -0.55254  0.08681  0.58827  1.91486
##
## Random effects:
##  Groups   Name        Variance Std.Dev.
##  Column   (Intercept) 12469    111.67
##  Residual              5618     74.95
## Number of obs: 36, groups:  Column, 4
##
## Fixed effects:
##             Estimate Std. Error       df t value Pr(>|t|)
## (Intercept) 1156.019    144.281   20.823   8.012 8.54e-08 ***
## EC          -165.136     80.198   29.385  -2.059 0.048442 *
## NO2          294.375    131.277   30.178   2.242 0.032434 *
## TimeValue    -11.108      2.667   30.783  -4.166 0.000233 ***
## WC           -23.065      6.130   25.708  -3.762 0.000878 ***
## ---
## Signif. codes:  0 '***' 0.001 '**' 0.01 '*' 0.05 '.' 0.1 ' ' 1
##
## Correlation of Fixed Effects:
##           (Intr) EC     NO2    TimeVl
## EC        -0.849
## NO2        0.143 -0.322
## TimeValue -0.549  0.363 -0.252
## WC        -0.617  0.549 -0.610  0.708
```

Supplementary File 4

# Correspondence analysis

Loading dataset

Replicate 1 and 2 from each dataset were loaded for a comparison between the dataset of unequal sizes. The dataset consists of soil chemical properties such as electrical conductivity, pH, water content, ammonium, nitrite and nitrate as explanatory variables and the species richness as a response variable.

Centering of variables and generating z-scores

In order to account for different dimensions of variables, they are centered transformed into z-scores. Centering was performed as subtracting variable means from its values and scaling was achieved by diving by variable's standard deviation.

```
df1$ECz = scale(df1$EC, center = TRUE, scale = TRUE)
df1$pHz = scale(df1$pH, center = TRUE, scale = TRUE)
df1$WCz = scale(df1$WC, center = TRUE, scale = TRUE)
df1$N.NH4z = scale(df1$N.NH4, center = TRUE, scale = TRUE)
df1$NO2z = scale(df1$NO2, center = TRUE, scale = TRUE)
df1$NO3z = scale(df1$NO3, center = TRUE, scale = TRUE)
```

Transfromed data visualisation

```
for (i in 1:6) {
    hist(df3[,i], xlim=c(-5, 5), breaks=seq(-5, 5, 0.2), main=colnames[i], probability=T
RUE, col="gray", border="white")
    d <- density(df3[,i])
    lines(d, col="red")
}
```

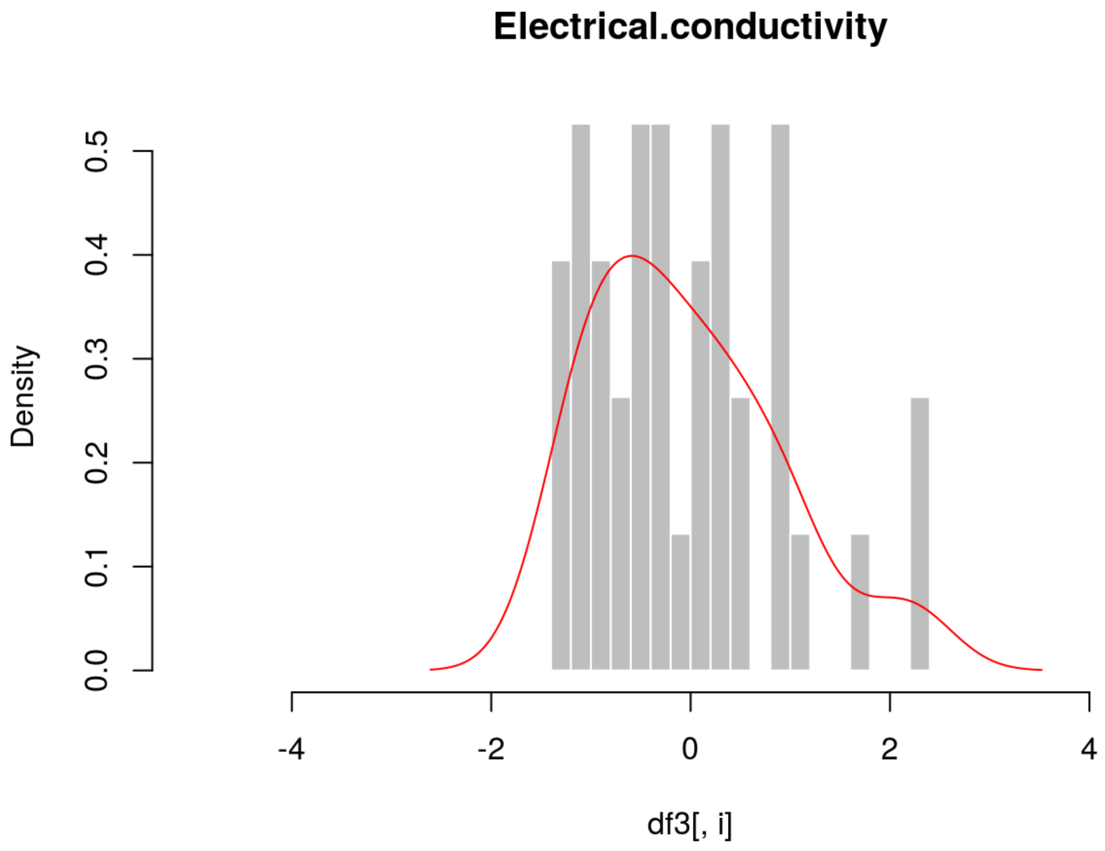
Electrical.conductivity
Density
0.0
0.1
0.2
0.3
0.4
0.5
-4
-2
0
2
4
df3[, i]

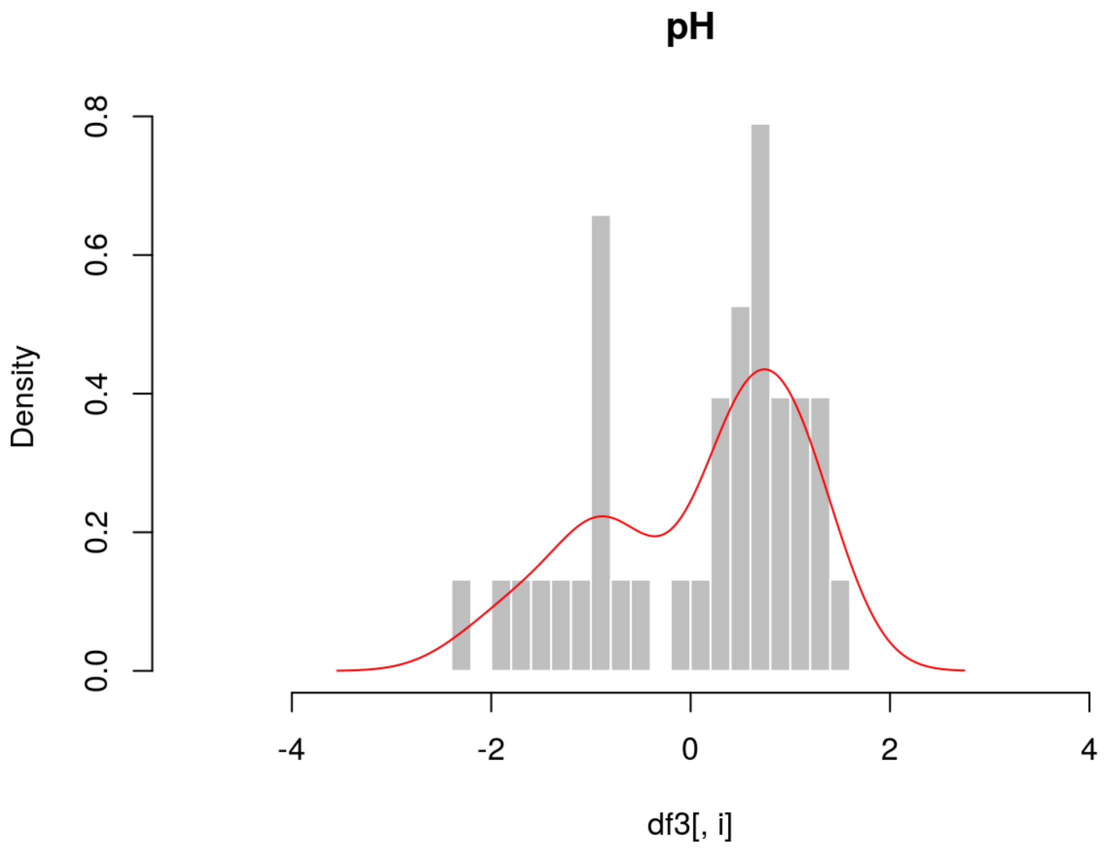
pH
Density
0.0
0.2
0.4
0.6
0.8
-4
-2
0
2
4
df3[, i]

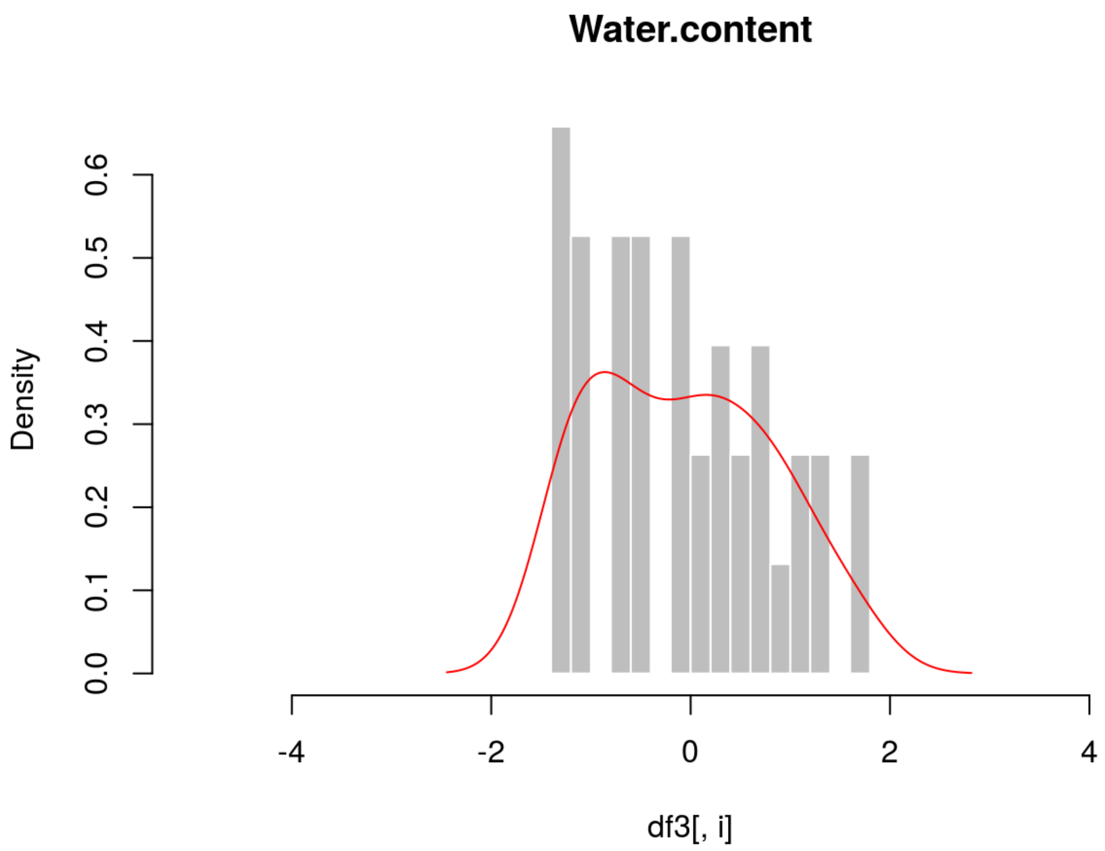
Water.content
Density
0.0
0.1
0.2
0.3
0.4
0.5
0.6
-4
-2
0
2
4
df3[, i]

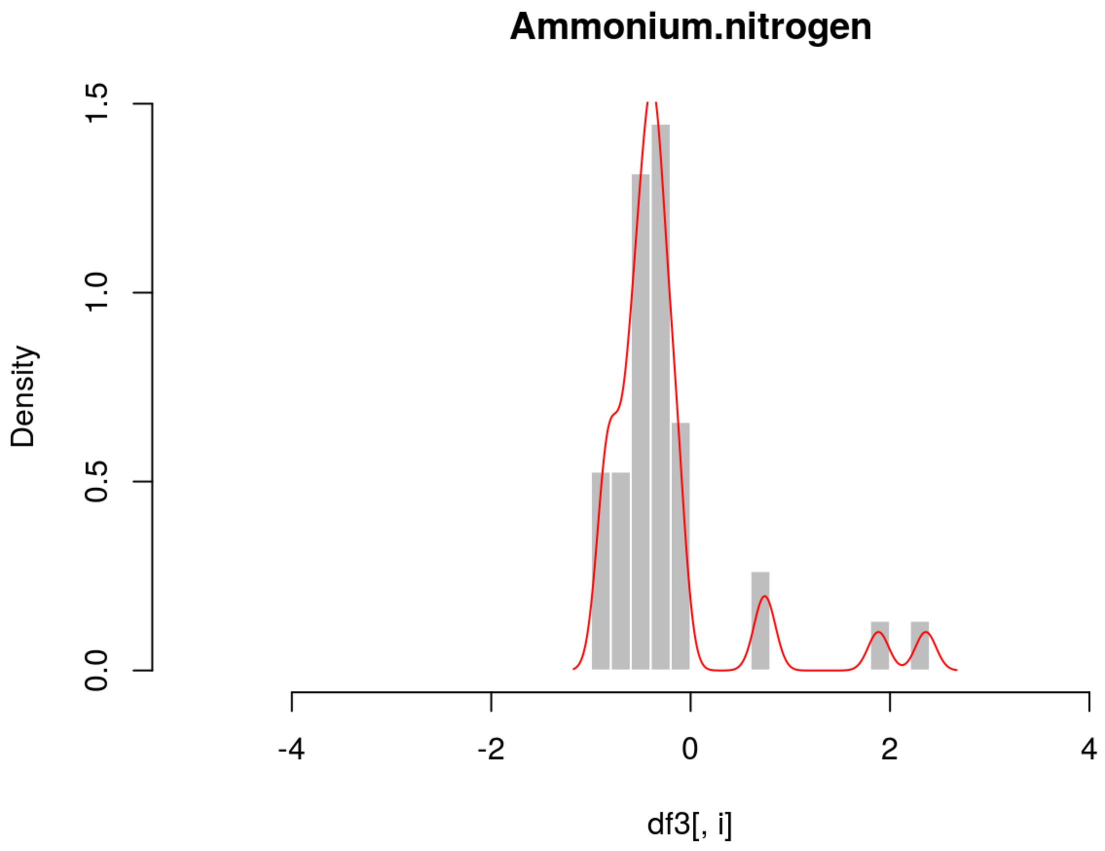
Ammonium.nitrogen
Density
0.0
0.5
1.0
1.5
-4
-2
0
2
4
df3[, i]

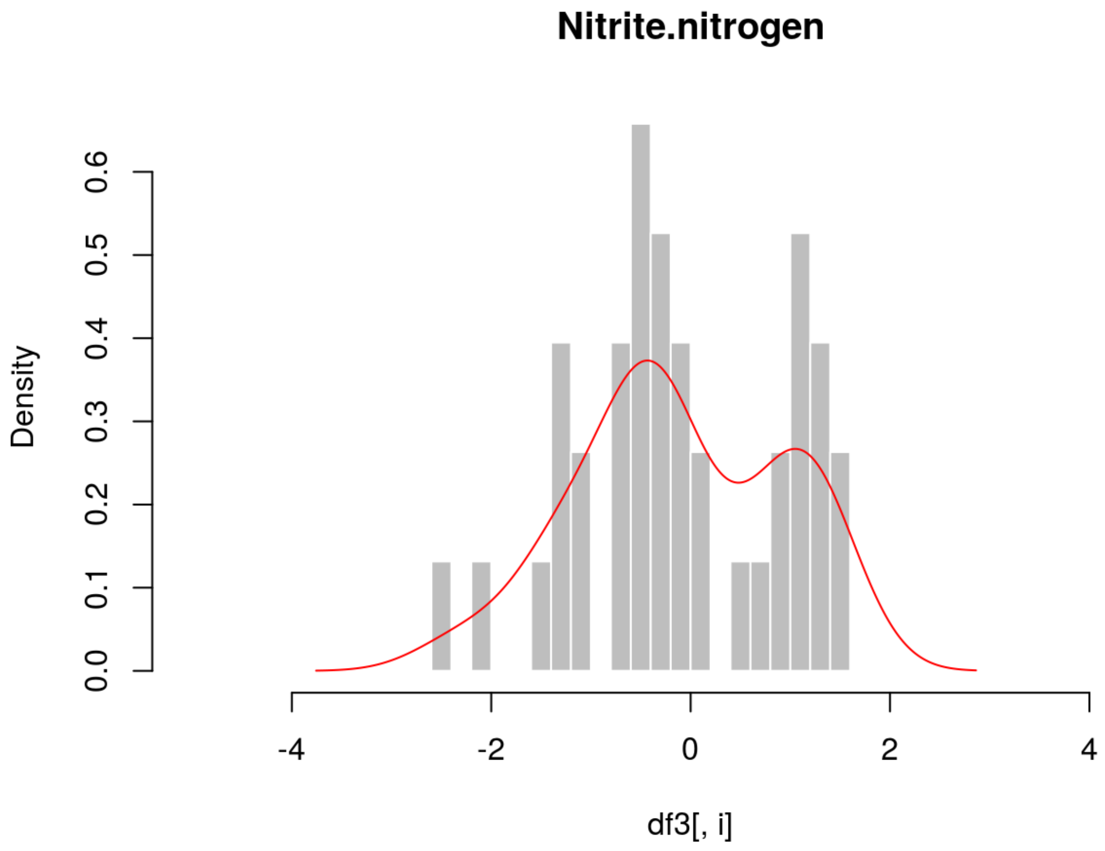
Nitrite.nitrogen
Density
0.0
0.1
0.2
0.3
0.4
0.5
0.6
-4
-2
0
2
4
df3[, i]

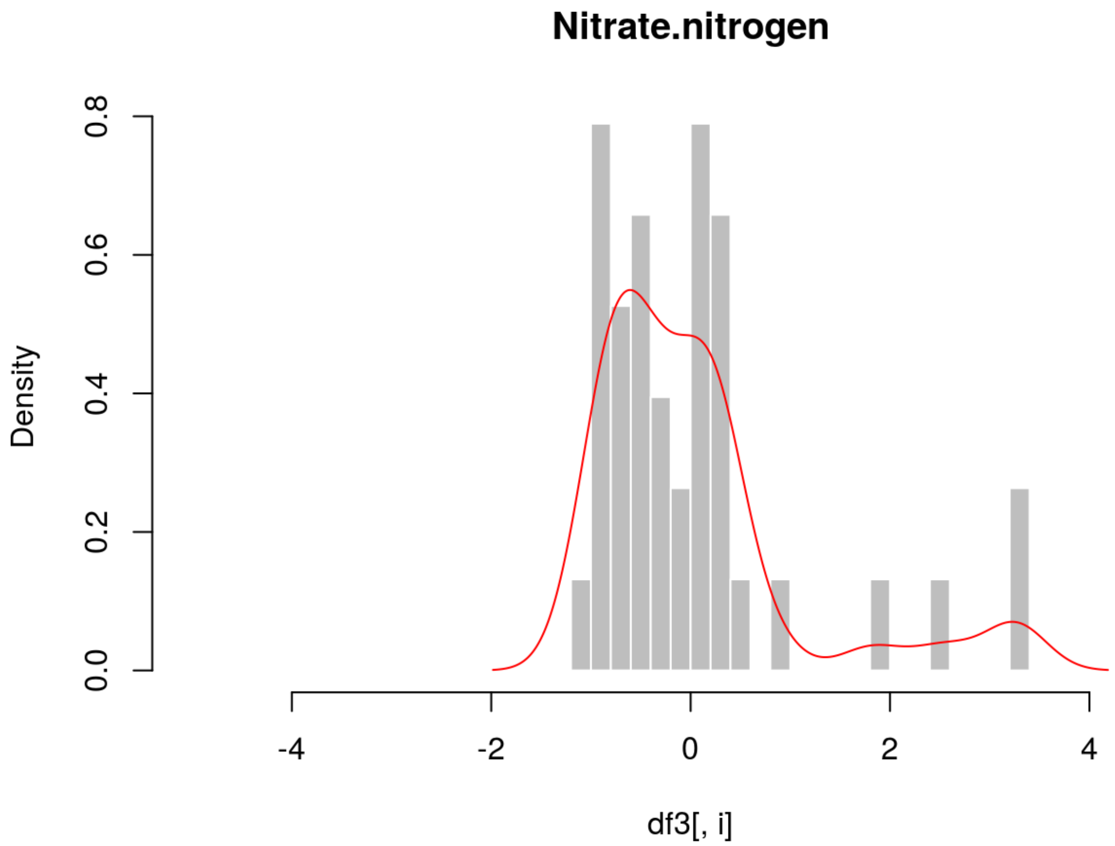

Full model statistics

```
ord <- cca(ani.otu ~ Time.value + Electrical.conductivity + pH + Water.content + Ammoniu
m.nitrogen + Nitrite.nitrogen + Nitrate.nitrogen, data=df4)

anova(ord, by = "term")
```

```
## Permutation test for cca under reduced model
## Terms added sequentially (first to last)
## Permutation: free
## Number of permutations: 999
##
## Model: cca(formula = ani.otu ~ Time.value + Electrical.conductivity + pH + Water.cont
ent + Ammonium.nitrogen + Nitrite.nitrogen + Nitrate.nitrogen, data = df4)
##                         Df ChiSquare      F Pr(>F)
## Time.value               1   0.03309 1.1699  0.248
## Electrical.conductivity  1   0.10588 3.7436  0.003 **
## pH                       1   0.03110 1.0996  0.291
## Water.content            1   0.17899 6.3284  0.001 ***
## Ammonium.nitrogen        1   0.12289 4.3447  0.002 **
## Nitrite.nitrogen         1   0.03012 1.0648  0.314
## Nitrate.nitrogen         1   0.03344 1.1823  0.250
## Residual                26   0.73539
## ---
## Signif. codes:  0 '***' 0.001 '**' 0.01 '*' 0.05 '.' 0.1 ' ' 1
```

Model simplification

Variables that do not have a significant effect have been removed. A resulting simplified model was checked again.

```
ord2 <- cca(ani.otu ~ Water.content + Electrical.conductivity + Ammonium.nitrogen,
data=df4)

anova(ord, ord2)
```

```
## Permutation tests for cca under reduced model
## Permutation: free
## Number of permutations: 999
##
## Model 1: c("ani.otu ~ Time.value + Electrical.conductivity + pH + Water.content + ",
 "    Ammonium.nitrogen + Nitrite.nitrogen + Nitrate.nitrogen")
## Model 2: ani.otu ~ Water.content + Electrical.conductivity + Ammonium.nitrogen
##   ResDf ResChiSquare Df ChiSquare      F Pr(>F)
## 1    26      0.73539
## 2    30      0.93610 -4  -0.20071 1.7741  0.015 *
## ---
## Signif. codes:  0 '***' 0.001 '**' 0.01 '*' 0.05 '.' 0.1 ' ' 1
```

The null hypothesis can be rejected and therefore the model is assumed significantly different from the full model. The environmental variable with the next highest significance is added to the model, in this cas it is the **time** since the start of the the analysis.

```
ord3 <- cca(ani.otu ~ Water.content + Ammonium.nitrogen + Time.value + Electrical.conduc
tivity, data=df4)

anova(ord, ord3)
```

```
## Permutation tests for cca under reduced model
## Permutation: free
## Number of permutations: 999
##
## Model 1: c("ani.otu ~ Time.value + Electrical.conductivity + pH + Water.content + ",
 "    Ammonium.nitrogen + Nitrite.nitrogen + Nitrate.nitrogen")
## Model 2: ani.otu ~ Water.content + Ammonium.nitrogen + Time.value + Electrical.conduc
tivity
##   ResDf ResChiSquare Df ChiSquare      F Pr(>F)
## 1    26      0.73539
## 2    29      0.84193 -3  -0.10654 1.2556  0.168
```

The null hypothesis can not be rejected and therefore it’s assumed that the simplified model is not statistically different from the original model and therefore it will be retained.

Final model

```
ord3
```

```
## Call: cca(formula = ani.otu ~ Water.content + Ammonium.nitrogen +
## Time.value + Electrical.conductivity, data = df4)
##
##               Inertia Proportion Rank
## Total          1.2709     1.0000
## Constrained    0.4290     0.3375    4
## Unconstrained  0.8419     0.6625   29
## Inertia is mean squared contingency coefficient
##
## Eigenvalues for constrained axes:
##   CCA1   CCA2   CCA3   CCA4
## 0.3326 0.0362 0.0334 0.0268
##
## Eigenvalues for unconstrained axes:
##     CA1     CA2     CA3     CA4     CA5     CA6     CA7     CA8
## 0.12955 0.07577 0.06449 0.05480 0.05090 0.04684 0.04442 0.04182
## (Showed only 8 of all 29 unconstrained eigenvalues)
```

```
anova(ord3, by = "term")
```

```
## Permutation test for cca under reduced model
## Terms added sequentially (first to last)
## Permutation: free
## Number of permutations: 999
##
## Model: cca(formula = ani.otu ~ Water.content + Ammonium.nitrogen + Time.value + Elect
rical.conductivity, data = df4)
##                         Df ChiSquare      F Pr(>F)
## Water.content            1   0.17328 5.9686  0.001 ***
## Ammonium.nitrogen        1   0.04499 1.5497  0.115
## Time.value               1   0.10073 3.4695  0.005 **
## Electrical.conductivity  1   0.10998 3.7882  0.001 ***
## Residual                29   0.84193
## ---
## Signif. codes:  0 '***' 0.001 '**' 0.01 '*' 0.05 '.' 0.1 ' ' 1
```

```
gg
```

It should be noted, that the CCA1 eigenvalue is much higher (0.3315) that the CCA2 eigenvalue (0.0356). This can be interpreted as CCA1 explaining proportionally ~ 9 times more variance that CCA2.

# Correspondence analysis assumptions

A high degree of environmental variable correlation has a strong impact on the data interpretation.

Environmental variables correlation matrix

```
corMat
```

```
##                          Water.content      N.NH4 Electrical.conductivity
## Water.content                1.0000000 -0.3567932              0.12181239
## N.NH4                       -0.3567932  1.0000000              0.28670049
## Electrical.conductivity      0.1218124  0.2867005              1.00000000
## Time                        -0.4533688  0.3923911              0.06790641
##                                 Time
## Water.content           -0.45336877
## N.NH4                    0.39239113
## Electrical.conductivity  0.06790641
## Time                     1.00000000
```

No strong correlation is identified between the variables.

Variance inflation factor

```
vif.cca(ord3)
```

```
##           Water.content       Ammonium.nitrogen              Time.value
##                1.410076                1.397673                1.367797
## Electrical.conductivity
##                1.165510
```

Variance inflation factor is sufficiently low for all the environmental variables.

Conclusion

The assumptions of the model have been met. The visual inspection and the ANOVA analysis support the main conclusion of the article. We can observe two major communities associated with either the dry (mostly purple crosses) or the wet samples (mostly turquoise crosses). Both water content and electrical conductivity are influencing the community structure along CCA1 axis, which is also by large margin the most explanatory. The CCA2 axis is mostly explained by the time of sampling. Different members of each of the two groups are dispersed more or less strongly along this axis, suggesting their early or late emergence.
